# Much More Than IL-17A: Cytokines of the IL-17 Family Between Microbiota and Cancer

**DOI:** 10.3389/fimmu.2020.565470

**Published:** 2020-11-10

**Authors:** Arianna Brevi, Laura Lucia Cogrossi, Giulia Grazia, Desirée Masciovecchio, Daniela Impellizzieri, Lucrezia Lacanfora, Matteo Grioni, Matteo Bellone

**Affiliations:** ^1^ Cellular Immunology Unit, Division of Immunology, Transplantation and Infectious Diseases, I.R.C.C.S. Ospedale San Raffaele, Milan, Italy; ^2^ Department of Medicine and Surgery, Vita-Salute San Raffaele University, Milan, Italy

**Keywords:** microbiota, Th17, autoimmunity, microbiome, gut, immunotherapy, arthritis, cancer

## Abstract

The interleukin-(IL-)17 family of cytokines is composed of six members named IL-17A, IL-17B, IL-17C, IL-17D, IL-17E, and IL-17F. IL-17A is the prototype of this family, and it was the first to be discovered and targeted in the clinic. IL-17A is essential for modulating the interplay between commensal microbes and epithelial cells at our borders (i.e., skin and mucosae), and yet, for protecting us from microbial invaders, thus preserving mucosal and skin integrity. Interactions between the microbiota and cells producing IL-17A have also been implicated in the pathogenesis of immune mediated inflammatory diseases and cancer. While interactions between microbiota and IL-17B-to-F have only partially been investigated, they are by no means less relevant. The cellular source of IL-17B-to-F, their main targets, and their function in homeostasis and disease distinguish IL-17B-to-F from IL-17A. Here, we intentionally overlook IL-17A, and we focus instead on the role of the other cytokines of the IL-17 family in the interplay between microbiota and epithelial cells that may contribute to cancer pathogenesis and immune surveillance. We also underscore differences and similarities between IL-17A and IL-17B-to-F in the microbiota-immunity-cancer axis, and we highlight therapeutic strategies that directly or indirectly target IL-17 cytokines in diseases.

## Introduction

Fine tuning of the interactions between eukaryotic and prokaryotic cells that literally share our body is essential for maintenance of health (1). In humans, the number of commensal, symbiont, and mutualistic microbes (i.e., microbiota) inhabiting the gut, skin, mucosae, and even some visceral organs, at least equals the number of eukaryotic cells (2). Nonetheless, the microbiome (i.e., the microbiota genomic repertoire) outnumbers the host’s genome by 10 folds (3), and this may help explaining why the microbiota is so relevant for the correct functioning of our organs and tissues (4). The development of individual microbiota starts soon after birth (5), and it stabilizes within the first three years (6). Within the same time frame, the developing immune system has to deal with, and it is shaped by the microbiota (7). Indeed, the immune system adapts to antigens expressed by eukaryotic cells, through the mechanisms of central and peripheral immune tolerance, thus avoiding autoimmunity (8). The immune system also has to progressively cope with antigens expressed by the microbiota (9), a phenomenon we originally defined as adaptation to the “extended self” (10). Tolerance to the extended self is likely enforced by the perfect balancing between regulatory T cells (Tregs), which block excessive immune reactions (11), and T helper (Th) cells, rapidly intervening when a commensal species has overtly grown, or a new species appears within the microbiota. Indeed, antigens and metabolites generated in the presence of a defined microbiota modulate the expansion or contraction of Tregs and effector Th cells (12–14). For example, microbiota-immune system interactions skew mouse Th cells to produce interleukin-17 (IL-17) (15), and together with IL-22 (16), Th17 cells producing these cytokines protect the integrity of the gut mucosa, and stimulate the local maturation of immunoglobulin (Ig) A-producing plasma cells, thus restraining dwelling bacteria (17). Additionally, fibroblasts, endothelial cells, chondrocytes, and adipocytes respond to IL-17A by expressing antimicrobial proteins and peptides, and proinflammatory cytokines and chemokines involved in acute-phase responses and tissue remodeling (18, 19). As a consequence, skin and mucosae of organisms lacking IL-17A are more susceptible to fungal and bacterial infection (20).

An alteration or imbalance of the normal microbiota composition (i.e., dysbiosis) is a common characteristic of many human diseases, albeit it remains to be clarified if dysbiosis is cause or consequence of the disease. Obesity, type 2 diabetes, nonalcoholic fatty liver disease, periodontitis, rheumatoid arthritis, psoriatic arthritis, multiple sclerosis, and systemic lupus erythematosus are examples of diseases exacerbated or worsened by an altered gut flora (1, 4, 10, 14, 21). Interestingly, IL-17A has a relevant pathogenic role in all these diseases (10). For example, it is well known that the IL-12-IL-17 axis exerts an essential role both in the onset phase and at the time of bone destruction in autoimmune arthritis (22). Microbiome analysis in rheumatoid arthritis patients showed dysbiosis and a relative abundance of *Prevotella copri*, Gram negative bacteria that appear to favor the induction of Th17 cells (23) (**Table 1**). In mice, transfer of Th17 cells polarized by *P. copri*-stimulated dendritic cells induced arthritis (38). Both in humans (39) and in mice (40), cross-reactivity between bacteria and myelin antigens seems to activate Th17 cells that induce autoimmune demyelination. In experimental autoimmune encephalomyelitis (EAE), microbiota-induced Th17 lymphocytes migrated from the gut into the central nervous system, where they exacerbated the disease (41). Thus, control of pathogenic Th17 cells occurs in the gut. The mechanisms by which commensals modulate the immune response, and Th17 cells in particular, has only been partially defined. Very recently, Duscha et al. (42) showed that the availability of propionic acid in feces and blood of multiple sclerosis patients depends on intestinal microbiota composition, and 14-day supplementation of propionic acid in the diet correlated with Treg expansion in the intestine, and neurologic symptom amelioration. Interestingly, monoclonal antibodies blocking either IL-17 or IL-23 are already in the clinic or under investigation for the treatment of rheumatoid arthritis patients (43). Thus, IL-17A is a master regulator of host-microbiota interactions both in physiologic conditions and in immune-mediated inflammatory diseases (44, 45).

**Table 1:** Microbes driving the production of IL-17 cytokines in inflammation and cancer.

	Microbes	Site	Cytokine produced	Producer cells	Outcome	Ref.
**Physiological inflammatory response**	*Tritrichomonas, Heligmosomoides polygyrus*	Intestine	IL-17E	Tuft cells	Activation of ILC2 and type-2 immunity in mice	(24)
	*Citrobacter rodentium*	Intestine	IL-17C IL-17B IL-17F	Epithelial cells	Induction of inflammation, promotion of epithelial barrier integrity in mice	(25–27),
	*Listeria monocytogenes*, *Influenza virus*	Intestine	IL-17D	Non-hematopoietic cells	Increased susceptibility to infection	(28)
**Inflammatory diseases**	*Pseudomonas aeruginosa*	Lungs	IL-17C	Epithelial cells	Induction of inflammation	(29)
	*Bacteroides stercoris*, *Bacteroides ovatus*, *Prevotella melaninogenica*	Lungs	IL-17B	Macrophages	Induction of pulmonary fibrosis in mice	(30)
	*Fusobacterium nucleatum*	Intestine	IL-17F	Epithelial cells	Correlates with progression of ulcerative colitis in humans and mice	(31)
	*Prevotella copri, Prevotella nigrescens*	Intestine	IL-17A	Th17	Correlates with enhanced rheumatoid arthritis in humans and mice	(32)
**Cancer**	*Proteobacteria, Verrucomicrobia*	Intestine	IL-17E	Macrophages	Correlates with progression of hepatocellular carcinoma in humans and mice	(33)
	*Escherichia coli*	Intestine	IL-17C	Epithelial cells	Colorectal cancer progression in mice	(34)
	Nontypeable *Haemophilus influenza*	Lungs	IL-17C	Epithelial cells	Progression of lung cancer in mice	(35)
	*Bacteroides fragilis*	Intestine	IL-17A	Th17 cells	Colorectal cancer progression in mice	(36)
	*Prevotella heparinolytica*	Intestine	IL-17A	Th17 cells	Multiple myeloma progression in mice	(37)

More recently, a microbiome has also been found in the blood and tumor of cancer patients (46), and microbiota-induced IL-17A has also been implicated in the pathogenesis of colon cancer, breast, pancreatic and ovarian carcinomas, and multiple myeloma (MM) (10, 47). The role of IL-17A in cancer has not been fully elucidated, and data are controversial. While in melanoma and ovarian cancer, Th17 cells activate anti-neoplastic cytotoxic T cell responses (48–50), they are tumorigenic in a variety of mouse models of colon cancer (51), hepatocellular carcinoma (52), MM (37), and pancreatic cancer (53). The function of IL-17 may also vary depending on the disease phase, and in pancreatic cancer it has been proposed that IL-17-producing cells support tumor growth in the initial phases of the disease, while in advanced phases, IL-17A potentiates antitumor immunity (47). IL-17A can favor tumor growth either in a direct or in indirect manner. In mouse tumor cell lines expressing the IL-17R, IL-17A induced IL-6 production, which in turn activated signal transducer and activator of transcription (Stat) 3, eventually upregulating pro-survival and proangiogenic signals (54). On the other hand, IL-17A also recruits mouse innate immune cells like neutrophils and immature myeloid cells within the tumor, supporting the development of an immunosuppressive microenvironment, eventually favoring tumor growth (55–57).

Of relevance, modulation of the gut microbiota reduces expansion of Th17 cells and tumor progression both in solid and hematopoietic tumors (37, 58). For example, in mice affected by MM (59), a neoplasia of plasma cells accumulating primarily in the bone marrow together with an immune infiltrate (60), the gut microbiota enriched in *P. heparinolytica* induced Th17 cells locally, which migrated to the bone marrow and promoted aggressiveness of MM (**Table 1**). Indeed, both in humans and in mice neoplastic plasma cells express the IL-17 receptor (IL-17R) (37, 61), and IL-17 supports plasma cells survival and proliferation likely by inducing the autocrine release of IL-6 (54). Lack of IL-17A in MM mice, or treatment with antibiotics or monoclonal antibodies blocking IL-17/IL-17R interactions delayed disease progression (37). Thus, the microbiota-IL-17A axis is also relevant in cancer patients.

The gut microbiota may also influence response to therapy in cancer patients, and this is the focus of intense clinical investigation. For instance, the composition of the gut microbiota *per se* is sufficient to discriminate cancer patients who will or will not respond to antibodies blocking inhibitory immune checkpoints (62–64). Prospective clinical trials will better define the impact of microbiota modulation on cancer therapy.

**Figure 1:**
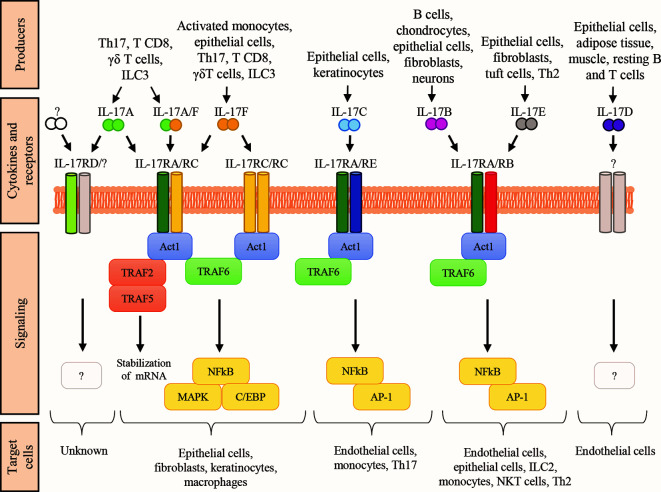
The IL-17 family of cytokines. Schematic representation of the cytokines belonging to the IL-17 family, their respective receptor complexes coupled with intracellular signaling, and their target cells. Cytokines are reported in a mechanistic rather than alphabetic order. Producers each cytokine are also shown. AP-1, activator protein-1; C/EBP, CCAAT enhancer-binding protein; ILC, innate lymphoid cells; MAPK, mitogen-activated protein kinase; NKT natural killer T cells; Th2, T helper-2 cells; Th17, T helper-17 cells; TRAF, TNF-receptor associated factor; NF-kB, nuclear factor kB.

IL-17A has been cloned in 1993 (65). At the beginning of this century, other molecules with sequence homology to IL-17A entered the IL-17 family, including IL-17B, IL-17C, IL-17D, IL-17E or IL-25, and IL-17F (66, 67). Each cytokine of the family acts as homodimer or heterodimer, and they interact with specific dimeric receptors (named IL-17RA, IL-17RB, IL-17RC, IL-17RD, and IL-17RE; **Figure 1**), with the exception of IL-17D, which remains orphan of its ligand (44). Binding of IL-17 cytokines to cognate IL-17Rs activates the shared SEFIR (SEF/IL-17R) cytoplasmic motif (68), which mediates the recruitment of Act1 (69). As detailed below, these steps are crucial for downstream recruitment and ubiquitination of TNF-receptor associated factor 6 (TRAF6), activation of nuclear factor κB (NF-κB), and expression of pro-inflammatory and anti-microbial molecules (70).

While the role of microbiota-driven IL-17A and Th17 cells in cancer have been extensively reviewed [e.g. (10, 20, 47)], a review dedicated to the role of the other cytokines of the IL-17 family in the microbiota-immunity-cancer axis is lacking. Thus, we intentionally overlooked the IL-17A/IL-17RA-RC pathway, and we have focused on IL-17B, IL-17C, IL-17D, IL-17E, and IL-17F.

## IL-17 Signaling

Cytokines of the IL-17 family are pleiotropic and exert potent and diverse *in vivo* functions through both canonical and noncanonical signaling pathways (68). Canonical signaling induces both transcriptional and post-transcriptional mechanisms involved in autoimmunity, hypersensitivity, and metabolic reprogramming of lymphoid tissues. Noncanonical signaling acts in synergy with other receptor systems, and it is mainly responsible for tissue repair and regeneration. Both mechanisms participate to host defenses, and tumor progression.

The IL-17Rs belong to a new subfamily of receptors consisting of 5 members: IL-17RA, IL-17RB, IL-17RC, IL-17RD, and IL-17RE which are single-pass transmembrane receptors with conserved domains (71) (**Figure 1**). Indeed, all members of the IL-17R family encode two extracellular fibronectin II-like domains and the conserved region SEFIR, which mediates the recruitment of the multifunctional adaptor Act1 (69). SEFIR is structurally related to the domain found in the toll-like receptor (TLR)/IL-1R (72). Functionally, the IL-17R is a heterodimeric complex composed of the IL-17RA in combination with other subunits that confer ligand or signaling specificity. IL-17A signals through IL-17RA in combination with IL-17RC. Whereas the IL-17RA subunit is ubiquitously expressed, IL17RC is mainly present on non-hematopoietic epithelial and mesenchymal cells. Interestingly, IL-17A interacts with its receptor as a homodimer or as a heterodimer with IL-17F (73). IL-17F could also bind this receptor complex as a homodimer. The difference between these three ligands is mainly in the potency of interaction: IL-17A>IL-17A-IL-17F> IL-17F (74). Also the IL-17RD has been proposed as an alternative receptor subunit for IL-17A, but not for IL-17F, and appears to favor the IL-17A-mediated recruitment of neutrophils (75). Finally, IL-17RA is also used by IL-17B, IL-17C, and IL-17E (also known as IL-25) (66). The detailed function of IL-17 receptors and their ligands remains partially elusive and requires further investigation.

### Canonical Signaling

The canonical IL-17 signaling pathway is initiated by SEFIR (**Figure 1**), which mediates Act1 recruitment (69). Act1 is crucial for IL-17 signaling, and it acts as adaptor and as RNA-binding protein (RBP) by forming several ribonucleoprotein particles (RNPs) (69, 76, 77). As adaptor, Act1 triggers multiple signaling cascades *via* the tumor TRAF-binding motif, which recruits different TRAF protein to initiate separate downstream pathways. The TRAF-binding motif is a distinct C-terminal region present only in IL-17RA. An analogous domain in other IL-17R family members is not found (68). Downstream recruitment and ubiquitination of TRAF6 leads to the activation of nuclear factor-kB (NF-kB), the CCAAT enhancer-binding proteins (C/EBPs) family, and the mitogen-activated protein kinase (MAPK) pathways (p38, ERK, and JNK) responsible for transcriptional regulation (44, 70). The TRAF6-mediated signaling is controlled by several regulatory mechanisms to hamper IL-17-induced inflammation. For instance, upregulation of IL-17 signaling *via* NFkB is associates with susceptibility to autoimmune syndromes, including psoriasis and experimental autoimmune encephalomyelitis (78, 79). Additionally, TRAF3 or TRAF4 compete with TRAF6 for the TRAF-binding motif on Act1, leading to reduced IL-17-induced expression of pro-inflammatory mediators, and Act1 is degraded by the proteasome in the presence of prolonged IL-17 stimulation (80). Thus, NF-κB and MAPK pathways downregulate IL-17 signaling. Conversely, C/EBP family activation potentiates the IL-17-inflammatory response through a feed-forward mechanism with other transcription factors like IκBζ. IκBζ modulation is crucial to control the IL-17-dependent responses, and it is one of the few targeted genes so far investigated. Indeed, most of the C/EBP-dependent genes involved in the IL-17 pathway remain elusive.

Act1 also acts as RBP upon TRAF2-TRAF5 complex engagement to control the stability and translation of mRNA from IL-17-target genes in response to IL-17 stimulation. IL-17 signaling results in the formation of multiple RNPs, associated with mRNA-stabilizing or mRNA-destabilizing factors, for post-transcriptional regulation of gene expression (81). Interestingly, IL-17 increases the half-life of mRNA to induce the efficient production of effector proteins.

### Noncanonical Signaling

Noncanonical IL-17 signaling is characterized by synergistic interactions of IL-17 signals with other ligands, like cytokines or microbial products, that lead to activation of diverse signaling pathways (82–84). As few examples, NF-kB is activated upon interaction of IL-17 with tumor necrosis factor-α (TNF-α) or lymphotoxin, and the signal transducer and activator of transcription 1 (STAT1) when IL-17 interacts with interferon-γ (IFN-γ). Interaction between IL-17 and IL-13 activates STAT6, whereas the SMADs family is triggered by the interaction with tumor growth factor-β (TGF-β). Finally, IL-17 interactions with bacterial lipopolysaccharide or fungal products, like candidalysin, activates c-Fos (82–84).

IL-17 also controls tissue homeostasis by integrating signals from the IL-17R and growth factor receptors in a high cell type- and context-specific manner. In particular, an integration of IL-17 receptor signaling has been described with the epidermal growth factor receptor (EGFR), the fibroblast growth factor receptor (FGFR), NOTCH1, and with components of the C-type lectin receptors. The EGFR cascade is mainly identified in skin stem cells, and it is involved in wound healing and tumorigenesis (85, 86). Interactions between IL-17 and FGFR have been described in mouse colonic epithelial cells during tissue repair caused by colon inflammation (87). In mice, IL-17 signaling also engages the NOTCH1 receptor to promote neuroinflammation through expansion and differentiation of oligodendrocyte progenitor cells (88, 89). Finally, signaling integrations between IL-17 and components of C-type lectin receptors have been reported in keratinocytes during psoriasis (90).

Altogether, these findings support the existence of a complex and yet partially explored net of signaling pathways downstream IL-17 cytokine secretion and interaction with their receptors.

## Cytokines of The IL-17 Family Other Than IL-17A in Health and Disease

Cytokines of the IL-17 family are crucial components of the inflammatory response, and they are essential for normal host immune responses. Both in humans and in mice, IL-17 cytokines are produced by a vast array of cell types, and act on a multitude of cellular targets (44, 66, 67, 91–93), eventually inducing production of pro-inflammatory cytokines, chemokines, and prostaglandins (94) (**Figure 1**). Cytokines of the IL17 family exert non-redundant, and even opposing functions to promote elimination of intruders, and tissue reconstitution. They are also involved in many human pathologies including inflammatory immune mediated diseases and cancer.

Cytokines within the IL-17 family share 16-50% amino acid identity with IL-17A, with IL-17F being the most similar (50%) and IL-17E the most divergent (16%). The similarity between IL-17 cytokines is higher in the C terminus and in five spatially conserved cysteine residues. N terminus sequences of IL-17B, IL-17C, and IL-17E are substantially different from those found in IL-17A and IL-17F because of a longer extension of the former three proteins (95), suggesting that the N terminus is involved in receptor specificity (96).

Because IL17F has the highest homology with IL-17A, binds to the same complex IL17RA-RC, and activated Th17 cells to produce both IL-17A and IL-17F (97), we followed a mechanistic rather than an alphabetic order to describe cytokines of the IL-17 family, and we started from IL-17F. As we will see, not all these cytokines are produced by immune cells, but all of them either directly or indirectly impact the immune system. We refer the interested reader to excellent reviews for a comprehensive description of these cytokines (44, 66, 67, 91–93).

### IL-17F

The *Il17f* gene is closely located to the *Il17a* gene both in humans (chromosome 6) and mice (chromosome 1), whereas genes encoding the other members of the IL-17 family are located in different chromosomes (18). The protein has a molecular mass of 18045 Da and is composed of 163 amino acids. IL-17F can form homodimer or heterodimer with IL17A (https://www.genecards.org/cgi-bin/carddisp.pl?gene=IL17F#summaries). Many genetic variants have been identified for the *Il17f* gene: most of them are missense mutations and some of them are pathogenetic (https://gnomad.broadinstitute.org/gene/ENSG00000112116?dataset=gnomad_r2_1). For example, the heterozygous missense mutation S95L (c.284C>T) in the *Il17f* gene has been found in patients with chronic mucocutaneous candidiasis, an infection caused by *Candida albicans* that affects nails, skin, and oral and genital mucosae. The S95L IL-17F mutant (IL-17FS95L) is normally expressed and forms homo- and heterodimers with IL-17F, IL-17FS95L, and IL-17A. However, IL-17FS95L is severely hypomorphic and exerts a dominant-negative effect by impairing the binding of its complexes to the receptor (98).

IL-17A and IL-17F are mainly produced by activated CD4^+^ T cells leading to the definition of a distinct T cell subset named Th17 (66). The differentiation of Th17 cells in humans is induced by several cytokines including IL-1β, IL-21, IL-23 and TGF-β that activate the Stat3- and the IRF4-dependent expression of retinoic acid receptor-related orphan receptor-γt (ROR-γt). Th17 cells comprise IL-17A and IL-17F double positive cells, but also populations that are only positive for IL-17A or IL-17F have been identified, suggesting that the mechanisms regulating IL-17A and IL-17F production are different. Interestingly, in mice the expression of Il17a but not Il17f is strictly coupled to the T cell receptor (TCR) signaling through the inducible T cell kinase (Itk)-mediated nuclear factor of activated T cells (NFAT) recruitment (99). These data demonstrate that in mice Itk specifically links TCR signaling to Il17a expression, thus regulating Th17 cell cytokines through NFATc1.

As for IL-17A, IL-17F is also expressed in innate lymphoid cells (ILCs), γδ T cells, natural killer T (NKT) cells and CD8^+^ T cells, but IL-17F is exclusively produced by activated monocytes and epithelial cells (25, 100, 101). Both IL-17A and IL-17F bind to the IL-17RA-RC heterodimer, and they induce a qualitatively but not quantitatively similar signal, being IL-17A far more potent than IL-17F. Both IL-17A and IL-17F can be secreted as disulfide-linked homodimers or heterodimers (18). Heterodimers exhibit intermediate levels of potency in inducing IL-6 and CXCL1 when compared to homodimeric cytokines (102).

**Figure 2:**
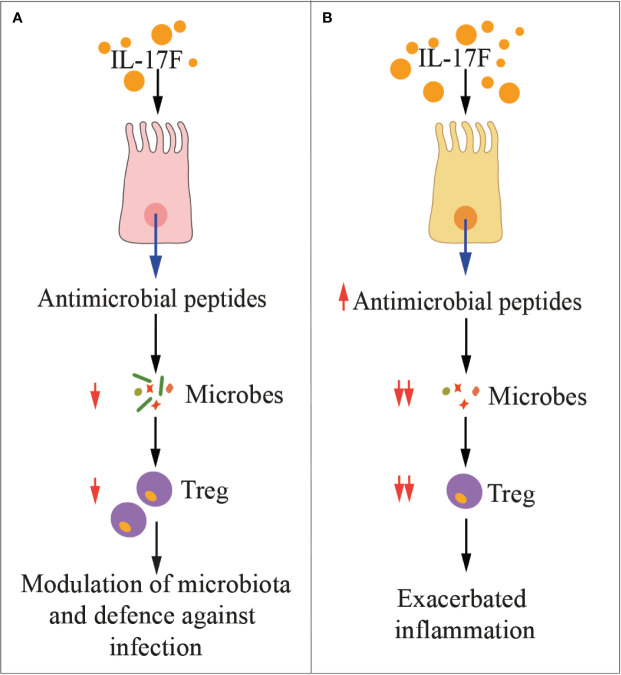
IL-17F in health and disease. The cartoon summarizes the main functions of IL-17F in physiologic conditions **(A)** and during inflammation **(B)**. Treg, regulatory T cells.

Both cytokines act in synergy with TNF-α (103), and in mice contribute to inflammation and protection at barrier surfaces, with overlapping yet distinct roles (**Figure 2**) (25). *In vitro*, IL-17F preferentially associates with IL-17RC homodimers, leading to IL-17RA-independent signaling (104). The expression profiles of IL-17RA and IL-17RC are different among tissues and cell types, with IL-17RC preferentially expressed in non-immune cells (25). In mouse models, the constitutive expression of IL-17RC in intestinal epithelial cells (25) explains the more pathogenic effects of IL-17F than IL-17A on microbiota during colitis (**Figure 2**) (105, 106).

### IL-17C

IL-17C is mainly known for its pro-inflammatory and antibacterial functions at epithelial sites in synergy with IL-17F (107). The *Il17c* gene is located in the long arm of human chromosome 16 (16q24.2). The IL-17C protein has a molecular mass of 21765 Da, and it is composed of 197 amino acids (https://www.genecards.org/cgi-bin/carddisp.pl?gene=IL17C). IL-17C share 23% amino acid homology with IL-17A (108), and while it binds a heterodimeric receptor formed by IL17RA and IL17RE, the IL-17RE subunit is the specific functional receptor for IL-17C (26). Most of the genetic variants of the *Il17c* gene are missense mutations (https://gnomad.broadinstitute.org/gene/ENSG00000124391?dataset=gnomad_r2_1), and just few of them have clinical significance (https://www.ncbi.nlm.nih.gov/clinvar/?term=il17c%5Bgene%5D).

**Figure 3:**
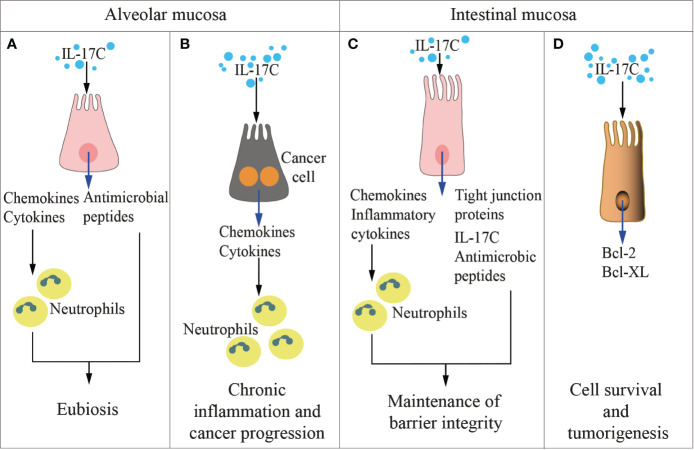
IL-17C in health and disease. The cartoon summarizes the functions of IL-17 C in the alveolar (left panels) and the intestinal (right panels) mucosae in physiologic conditions **(A, C)** and during inflammation and cancer development **(B, D)**.

Both in humans and in mice, the IL-17C is produced by several cells, including intestinal, tracheal and lung epithelial cells and keratinocytes, which also express the IL-17RA-RE heterodimer (107). Thus, IL-17C acts locally in an autocrine manner to protect the mucosa or to induce epithelial inflammatory responses (**Figure 3**) similarly to IL-17A and IL-17F (107). For example, stimulation of mouse epithelial cells by *Escherichia coli* or pathogen‐associated molecular patterns (PAMPs) activates a MyD88-dependent intracellular signaling, eventually inducing IL‐17C production, which activates expression of chemokines, granulocyte‐colony stimulating factor (GM-CSF), AMPs, and IL‐1β in an autocrine fashion (78). Additional target genes of IL-17C in epithelial cells encode antimicrobial peptides like S100A7/8/9, β-defensin2, immune-activating molecules CXCL1/2/3 and CCL20, and proinflammatory cytokines as well as occludin, claudin-1, and claudin-4, which are involved in the formation of epithelial tight junctions (107, 109). Interestingly, Wolf et al. showed that in mice, *Pseudomonas aeruginosa*‐induced IL‐17C expression in lung epithelial cells by a IL‐17A-dependent mechanism, thus demonstrating a network within the family of IL-17 cytokines that regulates each other expression (29).

IL-17C induces the expression of IL-1β and TNF-α in monocytes (67). IL-17RA-RE is also expressed on activated Th17 cells, and when triggered by IL-17C, it favors IL-17A, IL-17F, and IL-22 production by mouse Th17 cells, potentiating the adaptive immune response against pathogens and in autoimmunity (80, 110). Song et al. also identified the IL-17C/IL-17RE pathway as a pivotal regulator of innate immunity to intestinal bacterial pathogens in mice (26). Thus, IL-17C induces inflammation, but also promotes tissue healing.

IL-17C is involved in several human diseases. IL-17C levels are elevated in psoriatic lesions, and it significantly affects the abundance of F4/80^+^ macrophages within inflamed psoriatic plaques (107, 111, 112). Interestingly, IL-17C appears to also have a role in pathogenesis of atherosclerosis. Smith et al. reported that the mouse vasculature is an important source of IL-17C in atherosclerosis (113). Here, IL-17C exerts a pro-inflammatory role (114), by favoring the accumulation of pro-atherogenic Th17 cells within the aorta, which in turn affect the recruitment of monocytes and neutrophils to the plaque (115). Inflammatory glomerulonephritis also appears dependent on IL-17C, and Krohn et al. reported that patients affected by acute anti-neutrophil cytoplasmatic antibody-associated crescentic glomerulonephritis had significantly elevated serum levels of IL-17C (but not IL-17A, F, or E) (116). Additionally, they showed that glomerulonephritis ameliorated in mice lacking IL-17C and/or its receptor IL-17RE, and associated with a reduced Th7 response (116). We expect that in the next years IL-17C will be found involved in many more human diseases.

### IL-17B

The human *Il17b* gene was cloned together with *Il17c* and is located on the long arm of human chromosome 5 (5q32). The translated protein has a molecular mass of 20437 Da and is composed of 180 amino acids (https://www.genecards.org/cgi-bin/carddisp.pl?gene=IL17B). At the N terminus, there is an 18–20-amino acid sequence containing a hydrophobic motif, which functions as secretory signal sequence (117). IL-17B is secreted as a noncovalent dimer (18). Among the IL-17 family members, IL-17B has 29% homology with IL-17A (108).

Also for *Il17b*, most of the genetic variants are missense mutations (https://gnomad.broadinstitute.org/gene/ENSG00000127743?dataset=gnomad_r2_1). Almost nothing is known about their consequences, except for three specific conditions: a neurodevelopmental disorder of clinical uncertain significance, an hereditary cancer-predisposing syndrome and keratoconus, an eye condition that affects the shape of the cornea (https://www.disgenet.org/browser/1/1/2/27190/), and it is due to C176Y, C124Y protein changes. (https://www.ncbi.nlm.nih.gov/clinvar/?term=IL17b%5Bgene%5D).

IL-17B was found to be originally expressed in adult pancreas, small intestine, and stomach, but not in T cells (118, 119). IL-17B is also highly expressed in chondrocytes and neurons, although low IL-17B mRNA has been detected in several organs (120). According to recent investigations, IL-17B is weakly expressed by the epithelium, whereas IL-17B is strongly expressed in a healthy colon by connective tissue cells (121). IL-17B expression, especially in the epithelial and stromal compartments, is increased in colorectal cancer (121).

**Figure 4:**
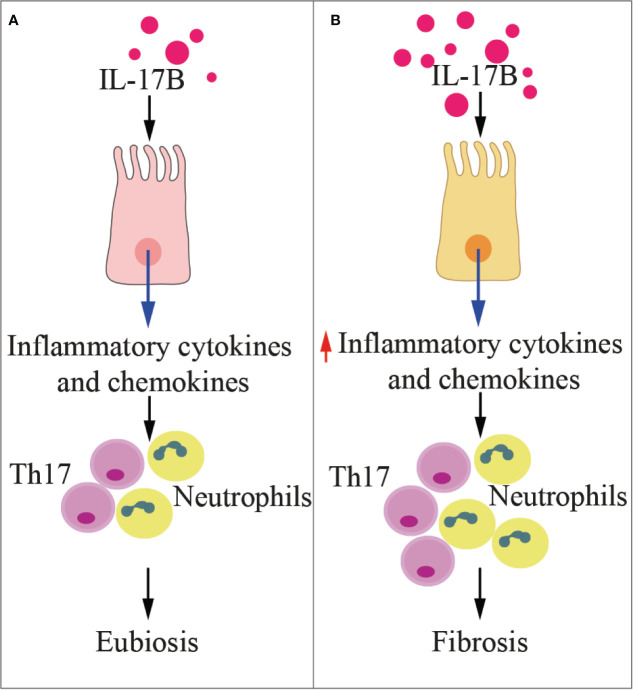
IL-17B in health and disease. The cartoon summarizes the functions of IL-17B in physiologic conditions **(A)** and during inflammation **(B)**. Th17, T helper 17 cells.

IL-17B and IL-17E (also named IL-25) share the same heterodimeric receptor IL-17RA/RB, and may exert redundant or contraposed effects, depending on the tissue context, as detailed below. The signaling pathway downstream IL-17RA-RB receptor is poorly detailed, and mainly described upon IL-17E binding. Li et al. showed that *in vitro* IL-17B does not induce IL-6 expression, but induces monocytes to produce TNF-α and IL-1β (118), and in mice it favors neutrophils recruitment (**Figure 4**) (119). *In vitro*, IL-17B promotes chemotaxis of IL-17RB-positive B cells by downregulating RGS16, the negative controller of CXCR4 and CXCR5 chemokine receptors (122). *In vivo*, IL-17B promotes embryonic development, tissue regeneration, and chemotaxis of B cells through IL-17RB in an autocrine fashion (93).

IL-17B has been investigated in several inflammatory diseases. While Ryan et al. (123) found genetic variants at the IL-17B locus in a 409 cases of coeliac disease and 355 controls, they did not find evidence that this locus was associated with the disease. Patients affected by systemic lupus erythematosus in the active phase showed higher levels of serum IL-17B than patients in the inactive phase (124). In community-acquired pneumonia, patients also showed higher serum levels of IL-17B when compared to healthy controls (125). IL-17B induced the expression of IL-8 in human bronchial epithelial cells through the activation of Akt, p38 mitogen-activated protein kinase, extracellular signal-regulated kinase (ERK), and NF-κB signaling pathways. Finally, in mice affected by pneumonia, high IL-17B levels significantly correlated with IL-8 concentrations (125). IL-17B also is the predominant cytokine of the IL-17 family in the rheumatoid synovia, it is locally produced by neutrophils, and it contributes to tissue destruction by enhancing TNF-α -induced production of G-CSF and IL-6 in fibroblasts (126). Interestingly, treatment with IL-17B neutralizing antibodies ameliorated collagen-induced arthritis in mice (127). Altogether, these findings demonstrate that IL-17B is a proinflammatory cytokine involved in inflammation and autoimmunity.

High levels of IL-17B have also been associated with poor prognosis in patients with pancreatic, lung or breast cancer, suggesting that the same signaling is exploited by cancer cells for survival, proliferation, and migration (93). Moreover, a relationship between IL-17B and stemness has been found in gastric cancer (128). Conversely, high levels of IL-17B appear to exert antiangiogenic activities *in vitro* (129).

### IL-17E

The *Il17e* gene is located on the long arm of human chromosome 14 (14q11.2). The molecular mass of the IL-17E protein, also named IL-25, is 20,330 Da, and it is composed of 177 amino acids. The IL-25 gene has two types of alternative spliced mRNA transcripts encoding two distinct subtypes (subtypes 1 and 2). Subtype 2 is different from subtype 1 for a shorter N end (130). To date, no studies have reported differences in the physiological role of the two subtypes. IL-17E shares only 17% homology with IL-17A, being the most distant among the cytokines of the IL-17 family (108). The human and mouse IL-17E genes share 80% homology (131). Genetic variants of *Il17e* gene are mostly missense mutations (https://gnomad.broadinstitute.org/gene/ENSG00000166090?dataset=gnomad_r2_1), and no specific clinical conditions have been associated to them (https://www.ncbi.nlm.nih.gov/clinvar/?term=IL25[gene]).

Intestinal tuft cells are the main producers of IL-25 (132). Additional sources of IL-17E exist, such as activated Th2 cells within the gastrointestinal tract and in other mucosal tissues (133), alveolar epithelial cells (134), alveolar macrophages (135), mesenchymal stem cells derived from the placenta and bone marrow (136), and mouse bone marrow-derived mast cells (137). IL-25 has been also found expressed in the murine central nervous system (138) as well as in the bronchial submucosa from asthmatic patients (139). In mice, IL-17E is also produced by brain capillary endothelial cells (140).

**Figure 5:**
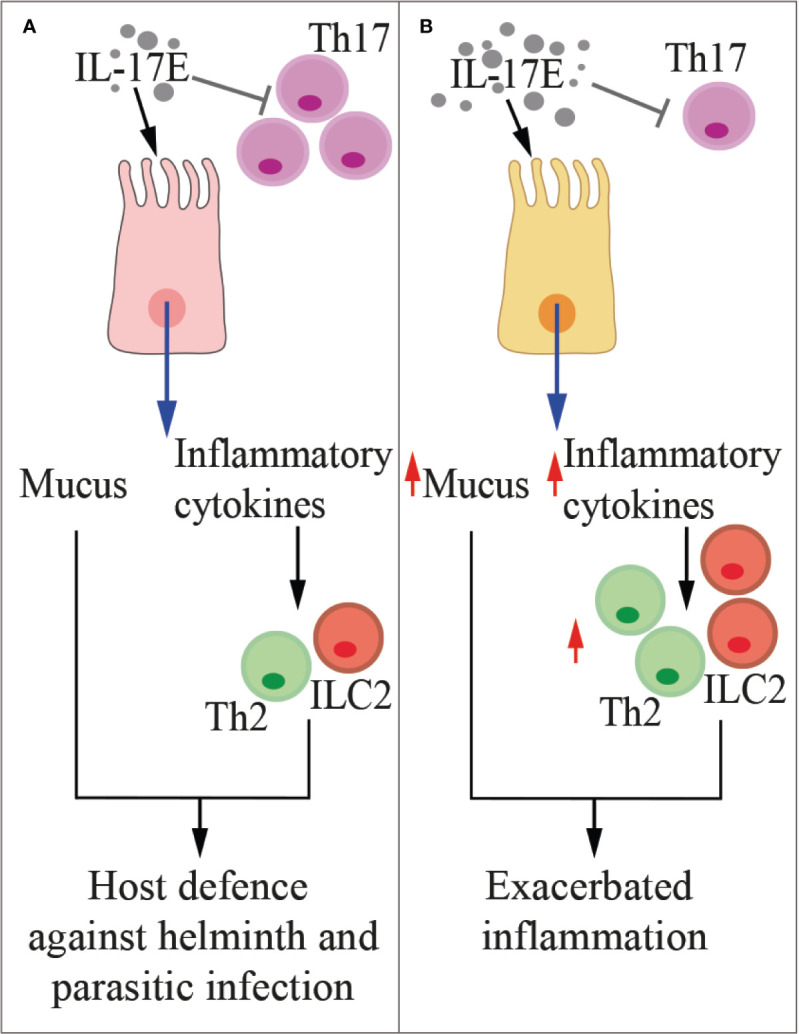
IL-17E in health and disease. The cartoon summarizes the functions of IL-17E in physiologic conditions **(A)** and during inflammation **(B)**. Th2, type-2 T helper cells, ILC2, type-2 innate lymphoid cells.

The receptor for IL-25 is composed of the IL-17RA and IL-17RB subunits (120). Thus IL-25 and IL-17B share the same receptor, and depending on tissue context, the two cytokines may exert redundant or contrasting effects. IL-17E stands among IL-17 family members for promoting the production of IL-4, IL-5 and IL-13 by innate type-2 immune cells (132, 141), nuocytes (142), T helper-2 cells, and NKT cells, thus contributing to the host defense against nematodes, but also to allergic reactions (133, 143). For example, after helminthic infection in mice, tuft cells-derived IL-17E induce ILC2 to produce IL-13, which activates epithelial cell progenitors resulting in the remodeling of the intestinal tissue and the induction of type-2 response (**Figure 5**) (132). Indeed, IL-17RA-RB triggering by IL-17E leads to TRAF6-mediated activation of NF-kB (144) and to the nuclear recruitment of the Th2 master regulator, GATA-3, in T cells (145). Additionally, IL-25 production is triggered in bronchial epithelial cells by rhinovirus infection, which causes local recruitment of eosinophils, neutrophils, basophils, and T and non-T type 2 cells, thus exacerbating asthma (146). IL-17E also amplifies a Th2 cell-dependent pathway in mice, thus promoting allergy (147).

While *in vitro* IL-17B elicits type 2 cytokine secretion (148), in several inflammatory conditions it antagonizes the pro-inflammatory activity of IL-17E by competing for the same receptor (27). IL-13 induced by IL-17E also inhibits IL-23, IL-1β, and IL-6 expression in activated DCs, thus blocking the induction of pathogenetic Th17 cells in autoimmune diseases (138). Interestingly, the IL-17E levels in the intestinal mucosa and serum of patients with active inflammatory bowel disease negatively correlated with endoscopic disease activity and C-reactive protein level (149), thus suggesting a protective role for IL-25 in this pathology. Indeed, IL-25 significantly inhibited the *in vitro* production of TNF-α, IFN-γ, and IL-17A by CD4^+^ T cells, but it promoted IL-10 secretion (149).

IL-17E appears to exert a dual role also in cancer. In a variety of human tumor xenograft models, including melanoma, breast, lung, colon, and pancreatic cancers IL-17E has an antitumor effect (150). However, IL-17E, likely released by epithelial tuft cells in the presence of intestinal dysbiosis, can promote the progression of hepatocellular carcinoma by favoring alternative activation of macrophages and their CXCL10 secretion in the tumor microenvironment (33). The role of IL-17E in tumors needs to be further investigated.

### IL-17D

IL-17D is the least investigated member of the IL-17 family (66). The *Il17d* gene is located on the long arm of the human chromosome 13 (13q12.11). The translated protein has a molecular mass of 21893 Da and is composed of 202 amino acids, making it the largest IL-17 (https://www.genecards.org/cgi-bin/carddisp.pl?gene=IL17D&keywords=il17d). Like all IL-17 family members, IL-17D also has four cysteine residues that may allow homodimer formation through interchain disulfide linkages (96). Whether it forms heterodimer is not known. IL-17D, unlike other members of the IL-17 family, shows an extended C-terminal domain, which may mediate a unique receptor interaction. Most *Il17d* genetic variants are missense mutations, but little is known about their phenotypes (https://gnomad.broadinstitute.org/gene/ENSG00000172458?dataset=gnomad_r2_1).

IL-17D was originally found to be highly expressed in skeletal muscle, adipose tissue, brain, heart, lung and pancreas (96). Curiously, resting CD4^+^ T cells and resting CD19^+^ B cell, but not activated T cells, express low levels of the cytokine, which is orphan of its receptor (96), although hints are coming from the sea lamprey. Investigators have found that IL-17D, which is the most expressed IL-17 in this ancient fish, interacts with IL-17RA in B-like cells (151).

IL-17D does not stimulate the proliferation of immune cells of its own, but, in response to stress, it induces endothelial cells to produce IL-6, IL-8 and GM-CSF (96). Recent studies have shown that IL-17D expression is regulated by the transcription factor nuclear factor erythroid-derived 2-like 2 (Nrf2), sensor for oxidative and xenobiotic stress (152). The Nrf2-mediated expression of IL-17D in response to carcinogenic stimuli initiates antitumor immune responses in mice by activating natural killer (NK)-mediated immune surveillance (152). IL-17D is required for optimal antiviral immunity as well: also in this case, viral infection induces Nrf2 and IL-17D, causing local oxidative stress and antiviral responses (152). Thus, IL-17D should contribute to protecting us from viruses and cancer. Whether IL-17D participates in immunity against other pathogens, such as intracellular bacteria, remains to be defined (153).

As for the other members of the IL-17 family, also IL-17D is implicated in autoimmunity. IL-17D RNA has been detected in rheumatoid nodules, where IL-17A is absent, but not in peripheral blood mononuclear cells or in synovial fluid from patients with rheumatoid arthritis (154). Conversely, IL-17D lacks in psoriatic skin (155), thus suggesting that the pathogenic mechanisms downstream IL-17D are heterogenous.

All together, these findings demonstrate that cytokines of the IL17 family exert non-redundant, and even opposing functions spanning from elimination of intruders or neoplastic cells and tissue reconstitution with limited collateral damage at the inflammation site, to pro-inflammatory and pro-tumoral activities.

## Cytokines of The IL-17 Family Other Than IL-17A and The Microbiota

As for IL-17A, also other cytokines of the IL-17 family are involved in maintaining homeostasis at the interface between microbiota and barrier epithelia (**Table 1**). In addition, overproduction of some of these cytokines may lead to immune mediated inflammatory diseases, and even propel cancer.

IL-17F is one of the major regulators of commensal microbiota in the intestine (**Figure 2**), where it is constitutively expressed and induces the production of antimicrobial peptides (i.e., defensins) (25). Whereas IL-17F appears to have a marginal pathogenic role in immune mediated inflammatory diseases, it exerts a crucial function in host defense against infections, as for example, against *Citrobacter rodentium* (25), a Gram negative enteropathogenic bacterium, which is equivalent of *E. coli* in humans. Defensins also extensively modulate the gut microbiota, and Tang et al. clearly showed that IL-17F-induced production of defensins constrained growth of commensal bacteria directly involved in the expansion of Tregs (105). As consequence, chemically-induced colitis was milder in mice deficient of IL-17F than that of IL-17A-deficient or wild type mice (105). Interestingly, in this experimental setting, IL-17A and IL-17F exerted opposing roles. Indeed, IL-17A was protective against colitis mainly by ensuring integrity of the gut mucosa, while IL-17F was proinflammatory. These experimental evidences have been validated in humans by showing that IL-17F RNA was elevated in colon biopsies from patients affected by ulcerative colitis, and together with IL-6 and TNF-α, they support the generation of a local inflammatory environment (31). Additionally, blockade of the IL-17A pathway in patients with bowel syndromes worsened the pathology (156). Thus, IL-17F may protect against pathogens, but also limit the local immunosuppressive activity of Tregs, eventually unleashing undesired inflammation.

Also the IL-17C is involved in maintenance of epithelial barrier integrity (**Figure 3**), where it is selectively induced by inflammatory or bacterial *noxae* (91). While IL-17C and IL-17A appear to exert overlapping functions (107), IL-17C is mostly produced by epithelial cells at very early time points, and acts both in autocrine and exocrine fashions by inducing the expression of tight junction proteins (109), proinflammatory cytokines and antimicrobial peptides (26, 107). On the contrary, IL-17A is also produced by immune cells like Th17 cells, γδ T cells, iNK T cells, macrophages, and ILCs. As an example, during infection with *C. rodentium*, IL-17C is upregulated in colon epithelial cells, and protects the mucosa in synergy with IL-22 (26), IL-17B (27), and IL-17F (25). IL-17C also attenuated inflammatory diseases like colitis, but it increased inflammation in psoriasis (107) and EAE (110) underlying the dual role exerted by this family of cytokines. At odds with IL-17A that controls fungal proliferation and infection, and whose blockage has been associated with fungal overgrowth and candidiasis (157), IL-17C is dispensable for immunity against candidiasis (158).

IL-17B is produced by epithelial cells in response to the abnormal expansion of pathobionts (i.e., commensals that in particular circumstances become pathogenic) within the microbiota (**Figure 3**). Its function is to protect the tissue and favor healing (93). Also in the course of allergic asthma, chemically-induced colitis or infection with *C. rodentium*, IL-17B exerts protective anti-inflammatory functions by interfering with IL-17E-induced IL-4 and IL-13 from type 2 Th cells and IL-6 from colon epithelial cells (27).

On the other hand, IL-17E, which also targets IL-17RA-RB, is essential for protecting the intestinal mucosa from parasitic infections (**Figure 4**) (159, 160). IL-17E production by tuft cells is constitutive and increases upon infection with natural mouse parasites like *Tritrichomonas* and *Heligmosomoid polygyrus*, resulting in stimulation of lamina propria ILC2 and mucosal tissue remodeling (**Table 1**). Schneider et al. (24) showed that *Tritrichomonas* favors fiber fermentation and intestinal accumulation of the short-chain fatty acid succinate, eventually inducing mouse intestinal tuft cells to release IL-17E, which in turn boosts type-2 immunity. Additionally, IL-17E produced by mouse intestinal epithelial cells upon microbiota stimulation limits the expansion of local Th17 cells (161) and IL-22 production by ILCs (162), thus identifying a delicate equilibrium among microbiota, adaptive immunity, and ILCs.

The expression of both IL-17E and IL-17B is upregulated during acute colonic inflammation (**Figures 4** and **5**), suggesting a dwelling activity between the two cytokines (27). Whereas IL-17B inhibits signaling of IL-17E but not of IL-17A or IL-17F, IL-17E does not interact with the IL-17RB homodimer, which remains available for IL-17B binding (27). Thus, the balance between IL-17B and IL-17E has to be fine-tuned to limit local inflammation and preserve mucosal integrity from the aggression of pathogens and pathobionts.

Also a dysregulated lung microbiota can drive IL-17B production, as it has been shown in a mouse model of bleomycin-induced lung fibrosis (30). More in details, the authors elegantly showed that depletion of the lung microbiota by antibiotics blocked bleomycin-induced lung fibrosis and death in mice. They also demonstrated that outer membrane vesicles locally released by *Bacteroides stercoris*, *B. ovatus*, and *Prevotella melaninogenica*, which were found enriched in the lung microbiota of mice treated with bleomycin, induced IL-17B production in the lung, thus favoring local immune cell infiltration and activation of profibrotic genes. These effects eventually increased bleomycin-induced mouse mortality. IL-17A and IL-17B have also been found in the bronchoalveolar lavage fluid of patients affected by lung fibrosis, but antibiotic treatments did not appear to be beneficial in limiting acute exacerbation in these patients (163, 164).

The role of IL-17D in the crosstalk between microbes and the immune system is less defined. Whereas IL-17D appears redundant in the context of inflammation induced by lipopolysaccharide, allergic agents or in EAE, it suppresses the function of DCs in inducing CD8^+^ T cell responses, thus favoring infection by *Listeria monocytogenes* or influenza virus (28). However, IL-17D also activates NK-mediated immune surveillance (152), thus potentiating innate immunity. Further investigation is required to clarify how these IL-17D-mediated mechanisms impact microbiota-host interactions.

## Cytokines of The IL-17 Family Other Than IL-17A, Microbiota, and Cancer

As for IL-17A, the role of IL-17B, IL-17C, IL-17D, IL-17E, and IL-17F in cancer remain controversial. While IL-17D and IL-17E appear to exert preponderant tumor-protective activities, IL-17B, IL-17C, and IL-17F are tumor promoting, either through a direct effect on tumor cells, or by modulating the tumor microenvironment.

### Anti-Tumor Activities of IL-17D and IL-17E

While IL-17D is released by several epithelial cells in response to pathogenic noxae, IL-17D expression in tumors does not appear compatible with their growth. In an elegant study, O’Sullivan et al. (165) showed that IL-17D derived from non-immunoedited cancer cells induces endothelial cells to produce monocyte chemoattractant protein 1 (MCP1), which is responsible for NK cell recruitment, eventually leading to tumor rejection. The same group also showed that mice deficient for IL-17D are more susceptible to viral infections and tumors (152). Nrf2 was shown to be responsible for IL-17D dependent recruitment of NK cells, and induction of Nrf2 by agonists led to regression of already established tumors *in vivo* (152). Thus, IL-17D might be an essential mechanism of immunoediting, and loss of IL-17D production might select for more aggressive tumors. Taking into account the propensity of IL-17A to propel tumor growth either in an autocrine (61, 166) or paracrine fashion (37), findings on IL-17D suggest that this cytokine counterbalances the pro-tumor activity of Th17 cells and IL-17A, and strategies to increase IL-17D might find clinical application. Gene expression analyses on human samples will define potential correlates between tumor immune infiltrate and expression of both IL-17A and IL-17D.

The potent pro-inflammatory activity of IL-17E also appears to be exploited against cancer. Purified IL-17E has been shown to delay growth of a variety of tumor xenografts when given alone or in combination with several drugs (150). The authors also documented accrual of eosinophils and activation of B cells in IL-17E-treated mice (150), but these mechanisms require further investigation.

IL-17E, which is also produced by mammary epithelial cells, has been shown to engage the IL-17RB on human mammary cancer cells, and to induce their caspase-dependent apoptosis. Interestingly this effect was restricted to neoplastic cells, because they express much more IL-17RB than normal mammary cells, and IL-17RB *in vivo* is expressed in high amounts in tumors from patients with poor prognosis (167). The authors also showed that purified IL-17E inhibited the growth of human mammary cancer cells xenografted in the mammary fat pad of mice (167). Additionally, administration of a synthetic compound able to induce IL-25 production by tumor associated fibroblasts suppressed growth of mammary tumor metastases in mice (168).

The effects of IL-17E might be context-dependent. It has been reported that the addition of cisplatin to cervical cancer cell cultures induced IL-17E and IL-17RB down-regulation, eventually inhibiting *in vitro* growth, migration, and invasion (169), Thus, IL-17E might exert a tumor-promoting activity, unless the latter depends on IL-17B, which also interacts with IL-17RB. *In vivo* data in genetically modified mice will clarify the effect of the two cytokines in cervical cancer.

### Pro-Tumor Activities of IL-17B, IL-17C, and IL-17F

IL-17B acts as tumor promoter in several solid and hematopoietic malignancies (**Figure 4**). Furuta et al. showed that the IL-17B/IL-17RB signaling is critical for breast tumorigenesis, and that IL-17RB expression correlates with poor prognosis in breast cancer patients (167). Engagement of IL-17B with its receptor induces Nf-kB-mediated upregulation of Bcl-2 expression, and resistance of mammary cancer cells to etoposide (170). Because IL-17B and IL-17E share the same receptor heterodimer, and IL-17E induces apoptosis in mammary cancer cells (167), an opposing role for IL-17B and IL-17E can be hypothesized in breast cancer. It will be necessary to understand how two similar cytokines engaging the same receptor deliver anti- or pro-apoptotic signals.

Up-regulation of IL-17RB expression was also found in pancreatic cancer, where expression of IL-17RB associated with metastasis incidence and reduced progression free survival (171). IL-17RB triggering induced CCL20/CXCL1/IL-8/TFF1 chemokine expressions *via* the ERK1/2 pathway, thus promoting macrophage and endothelial cell recruitment at primary sites, cancer cell invasion and survival at distant sites. *In vivo*, anti-IL-17RB monoclonal antibodies inhibited tumor metastasis and prolonged survival in a mouse xenograft model (171). Others confirmed a direct tumor-promoting activity of IL-17B in gastric cancer (172), thyroid cancer (173), and in acute myeloid leukemia (174).

A direct connection between local microbiota, cytokine production and tumorigenesis has been reported for IL-17C (**Figure 3**). Song et al. found that IL-17C is upregulated in human colorectal cancers (34), and alterations in the microbiota (**Table 1**) drove IL-17C upregulation specifically in murine intestinal epithelial cells, eventually supporting their survival and neoplastic transformation (34). In line with these findings, it has been reported that both intra- and peri-tumoral expression of IL-17RE predict early and late recurrence in hepatocellular carcinoma (175).

IL-17C, which promotes neutrophilic inflammation (**Figure 3**), was also found abundant in human lung cancer samples, and IL-17C is a negative prognostic factor in patients with lymph node metastasis (35). Patients with chronic obstructive pulmonary disease are highly susceptible to non-small cell lung cancer, and often harbor IL-17C-inducing nontypeable *Haemophilus influenza* in their lungs (**Table 1**). In IL-17C-deficient mice, nontypeable *Haemophilus influenza* induced less neutrophil lung infiltrates and promoted less tumorigenesis (35), thus linking IL-17C to bacteria and lung cancer.

IL-17A and IL-17F share the same heterodimeric receptor (IL-17RA-RC). Tang et al. showed that mice deficient for IL-17F, and not mice deficient for IL-17A, resist chemically induced colitis, and this correlates with a different gut microbiota (105). *Fusobacterium nucleatum*, which has been linked to chronic inflammation and cancer (176), aggravates intestinal inflammation in mice by targeting caspase activation and recruitment domain 3 through NOD2, eventually activating the IL-17F/NF-κB pathway (31). Because colitis often anticipates colon cancer, a microbiota-modulated, tumor promoting role for IL-17F can be hypothesized, and it needs to be proven in *in vivo* experimental settings.

Strong correlations have been found between IL-17RA, microbiota, and cancer, and most of them have been attributed to IL-17A. As IL-17B, IL-17C, IL-17E, and IL-17F also exploit the subunit IL-17RA to deliver their intracellular signals, mice selectively deficient for either these cytokines or the IL-RC, IL-RB, and IL-RE will help in better understating the role of the different cytokines of the IL-17 family in the microbiota-immunity-cancer axis.

## Strategies to Target Cytokines of The IL-17 Family

Several strategies are being adopted in the clinic to impact the microbiota-IL-17 axis (**Table 2**). They include diets, prebiotics, probiotics or even fecal microbiota transplantation in effort to transiently or permanently modify the microbiota and eventually the immune response. Additionally, monoclonal antibodies directed against IL-17A or other cytokines and receptors of the IL-17 family are used or are under investigation. These strategies are tested both in inflammatory diseases (21, 178–182) and cancer (183–185).

**Table 2:** Therapeutic strategies under investigation to target cytokines of the IL-17 family.

Therapeutic Agent	Target Molecule	Impact on disease	Clinical Trial Number/Ref.
**Brodalumab**	IL-17RA	Reduced symptoms in rheumatoid arthritis and psoriatic arthritis patients	NCT00771030 NCT01059448 NCT00950989 NCT02024646 NCT02029495 NCT04183881 NCT01516957
**Bimekizumab**	IL-17A- IL-17F	Reduced symptoms in psoriatic arthritis patients Reduced chemical-induced colitis in mice	NCT02969525 (105)
**Anti-IL-17RB**	IL-17RB	Delayed pancreatic tumor growth and metastasis formation in mice	(171)
**MOR106**	IL-17C	Reduced atopic dermatitis in mice Ineffective against human atopic dermatitis	(177) NCT03864627 NCT03568071 NCT03689829 NCT02739009
**Antibiotics**	↓ IL-17B ↓ IL-17C ↓ IL-17F	Reduced bleomycin-induced lung fibrosis in mice Reduced colon cancer formation in mice Reduced chemical-induced colitis in mice	(30, 34, 105)
**Q2-3**	↑ IL-17E	Reduced breast cancer metastasis in mice	(168)
**tBHQ**	↑ IL-17D	Delayed growth of B16 melanoma, Burkitt’s lymphoma and MCA-induced sarcoma in mice	(152)

Brodalumab, fully human IgG2 monoclonal antibody against IL-17RA; Bimekizumab, humanized IgG1 monoclonal antibody against both IL-17A and IL-17; MOR106, fully human IgG1 monoclonal antibody against IL-17C; Q2–3, synthetic dihydrobenzofuran lignan; tBHQ, Tert-butylhydroquinone; MCA, methylcholanthrene.

In the field of cancer, almost 200 clinical trials are ongoing that aim either at identifying the microbiota accompanying malignancies, or at testing microbiota-modulating strategies. The composition of the gut microbiome is being analyzed in breast cancer (NCT03885648), colorectal cancer (NCT03385213), lung cancer (NCT04333004), thyroid cancer (NCT03543891), hepatocellular carcinoma (NCT02599909), and glioblastoma (NCT03631823) among others. There is also interest for the microbiota of the lung in lung cancer (NCT03068663), of the oronasal cavities in hematopoietic malignancies (NCT02949427), or even the intratumor microbiota as for breast cancer (NCT03586297) and prostate cancer (NCT03947515). Several clinical trials are designed to modify the microbiota and increase susceptibility to chemotherapy (NCT04138979), radiotherapy (NCT02559349), or immunotherapy (NCT04116775). A more extensive list of clinical trials on this issue is out of the scope of this review, and can be found at (www.clinicaltrials.gov). Many of them interfere with the interplay between microbiota and the immune system, thus impacting all the cytokines of the IL-17 family.

Monoclonal antibodies against IL-17A or IL-17RA are already available to patients affected by psoriasis and arthritis (45), and might even find application in malignancies (10, 47). Results from one clinical trial with anti-IL17A monoclonal antibodies in MM patients are longed for (NCT03111992). Brodalumab, a monoclonal antibody against IL-17RA is also under investigation in patients affected by rheumatoid and psoriatic arthritis (**Table 2**), and it might also find application in cancer patients.

Few approaches have instead been proposed to target cytokines of the IL-17 family other than IL-17A. Because IL-17F can be tumor promoting (31), it will be interesting to investigate the anti-tumor activity of Bimekizumab especially in colorectal cancer (105) (**Table 2**). Bimekizumab is a monoclonal antibody against both IL17A and IL-17F, which is currently investigated in psoriatic arthritis patients (186). Anti-IL17RB monoclonal antibodies might impact metastatic pancreatic cancer, has it has been shown in a mouse model (171). IL-17C is an interesting target in colorectal cancer because IL-17C has been found upregulated in these tumors, and in mice IL-17C was modulated by the gut microbiota (34). MOR106 is a humanized monoclonal IgG1 antibody against IL-17C, which has been developed to treat atopic dermatitis (177). Unfortunately, the clinical development program of MOR106 in atopic dermatitis was ended because of disappointing results. Because blocking IL-17C signaling significantly reduces the number and extension of colonic tumors in mice, MOR106 might be investigated in human colorectal and lung cancer (35). MOR106 would be of advantage in respect to anti-IL17A because IL-17C/IL-17RE signaling is dispensable for immunity to systemic, oral, and cutaneous candidiasis (158). Thus, either blocking the IL-17C/IL-17RE axis or acting on the gut microbiome might be beneficial to cancer patients (**Table 2**).

Q2-3, a synthetic dihydrobenzofuran lignan that stimulates production of IL-25, which competes with IL-17B for the IL-17RB receptor, reduces myeloid derived suppressor cell infiltration and metastasis appearance in a mouse model of breast cancer (168), suggesting its potential application to prevent breast cancer metastasis in humans (**Table 2**). Nonetheless, targeting the IL-17RB in cancer should be carefully investigated in breast cancer, because it could interfere with the anti-tumor activity of IL-17E (167).

Finally, Nrf2, a cellular checkpoint of xenobiotic and oxidative stress (187) is an interesting molecule, as it delays tumor growth by stimulating IL-17D production in tumor cells, which recruits NK cells within the tumor (**Table 2**) (152). An advantage of such compound is that it activates the tumor autocrine Nrf2/IL-17D signaling, by inducing cellular stress without producing reactive oxygen species. As an example, Tert-butylhydroquinone (tBHQ) has been tested in preclinical models of B16 melanoma, human Burkitt’s lymphoma, and in the MCA-induced sarcoma, where activated Nrf2 and IL-17D production, resulting in delayed tumor progression (152). Nrf2 agonist are currently in clinical trials (e.g., NCT03182959, and NCT03934905).

## Conclusions and Perspectives

**Figure 6:**
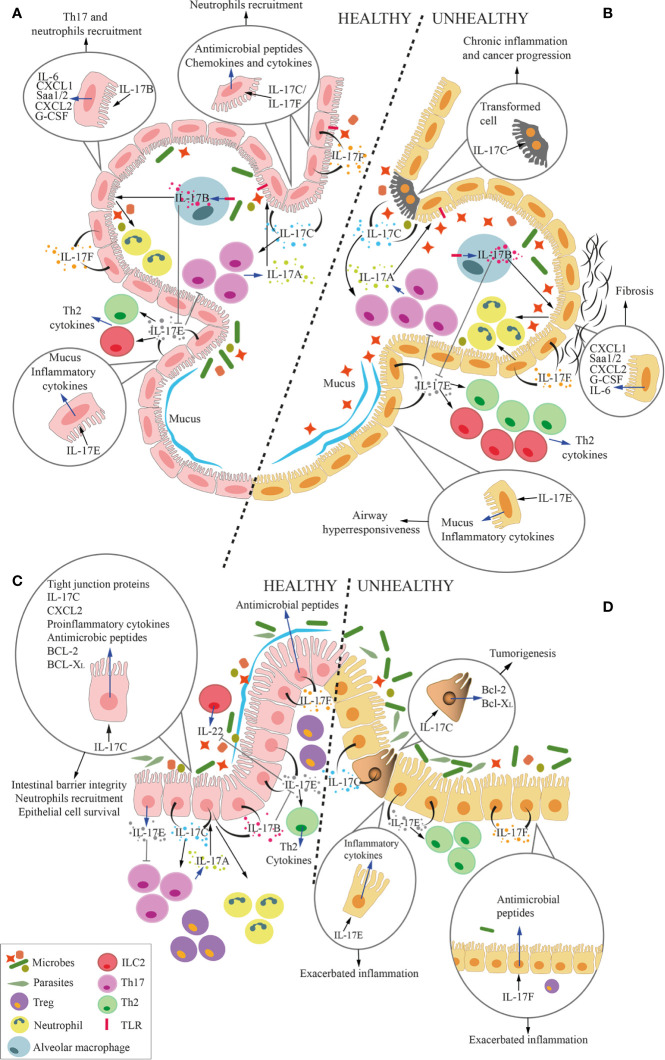
Overall function of IL-17 cytokines at the microbiota-host interface in the lung and the gut. Cartoon summarizing the overall role of IL-17 cytokines at the interface between microbiota and alveolar **(A, B)** and intestinal mucosa **(C, D)** in health **(A, C)** and disease **(B, D)**. Circles within the panels enlarge and focus on several effects of IL-17 cytokines on epithelial cells. Blue arrows represent the secretion of cytokines or the expression of genes by cells, whereas black arrows represent the stimulation of the cell by the cytokines. **(A, C)** In physiologic conditions, IL-17A, which is mostly released by Th17 cells, keep growth of commensal microbes residing in the lumen of the respiratory tract or the intestine under control. Mucosal epithelial cells secrete IL-17B, IL-17C, IL-17E and IL-17F in response to stimuli coming from the local microbiota. More in details, IL-17B, produced by alveolar macrophages (MØs) under TLR4-mediated stimulation, act on epithelial cells inducing release of several factors, among which IL-6, serum amyloid A1 and A2 (Saa1/2), CXCL1, CXCL2, and G-CSF. Some of these factors favor local recruitment of Th17 cells and neutrophils, which also contribute to maintain an adequate balance in the microbiota composition. Additionally, IL-17B induces monocytes to release TNF-α and IL-1β, which also favor neutrophil recruitment (not shown). IL-17C and IL-17F are released by epithelial cells, and act in autocrine and paracrine fashion inducing the production of antimicrobial peptides, but also chemokines and cytokines that favor neutrophil recruitment. IL-17C also promotes Th17 cell responses, and it supports barrier integrity through tight junction formation in epithelial cells. Also IL-17C induces expression of TNF-α and IL-1β in monocytes (not shown). IL-17E favors the induction of type 2 responses by Th2 cells and ILC2, whereas IL-17B blocks this action, thus avoiding excessive type 2 immune responses. While IL-17D activates NK-mediated immune surveillance (not shown), its relationship with lung and gut microbiota remains unknown. Therefore, IL-17D is not depicted in the figure. IL17E inhibits IL-23, IL-1β and IL-6 expression in activated dendritic cells (not shown), thus blocking the induction of pathogenic Th17 cells. Healthy alveolar epithelial cells also secrete mucus in response to IL-17E to protect the epithelium from bacterial adhesion. **(B, D)** In pathologic conditions, excessive IL-17A causes local inflammation. In response to the expansion of pathobionts, MØs release more IL-17B, which acts on epithelial cells to induce pro-inflammatory signals (IL-6, Saa1/2, CXCL1, CXCL2, and G-CSF), which may induce lung fibrosis. Stimuli from pathogenic bacteria unleash IL-17C hyperproduction, leading to chronic inflammation and tumorigenesis, also through the upregulation of Bcl-2 and Bcl-XL. Excessive IL-17E signaling is associated with stronger Type 2 immune reaction (Th2 and ILC2) that exacerbate airways hyperresponsiveness and gut inflammation. Unbalanced IL-17F in the gut induces the release of excessive antimicrobial peptides, which constrains Treg-inducing bacteria, therefore promoting gut inflammation.

While characterized by a common genetic origin, cytokines from the IL-17 family demonstrate a wide heterogeneity in functions as well as in cellular source, and kinetic of production and secretion (**Figure 6**).

An intriguing evidence is that even if some of these cytokines share the same receptor, they may exert opposite downstream activities. For instance, blocking IL-17A is detrimental rather than curative in the murine model of chemically-induced colitis, and blockage of IL-17F either alone or with IL-17A resulted in disease amelioration (105). Thus, blocking IL-17RA impacts all IL-17 cytokines but IL-17D, and might exert unpredictable/undesired effects.

The same unpredictable/undesired effects might occur when attempting to modulate the microbiome. Examples are available of unexpected side effects of patients treated with probiotics (181).

All together these findings suggest that even if very fascinating and promising, the actual knowledge on the role of IL-17 cytokines in cancer is preliminary. A plethora of information about these cytokines in health and disease is waiting to be unveiled in next years.

## Author Contributions

AB and MB developed the concept of the review. All authors participated to retrieve the relevant literature, wrote and prepared the manuscript. DM and LC prepared the figures. All authors contributed to the article and approved the submitted version.

## Funding

The work was supported by Associazione Italiana per la Ricerca sul Cancro (AIRC; grant #IG21808 to MB). AB was supported by a fellowship from the Fondazione Italiana per la Ricerca sul Cancro/AIRC (grant #22316).

## Conflict of Interest

The authors declare that the research was conducted in the absence of any commercial or financial relationships that could be construed as a potential conflict of interest.

## References

[1] Lynch SV Pedersen O The Human Intestinal Microbiome in Health and Disease. N Engl J Med (2016) 375:2369–79. 10.1056/NEJMra1600266 27974040

[2] Sender R Fuchs S Milo R Are We Really Vastly Outnumbered? Revisiting the Ratio of Bacterial to Host Cells in Humans. Cell (2016) 164(3):337–40. 10.1016/j.cell.2016.01.013 26824647

[3] Ley RE Peterson DA Gordon JI Ecological and evolutionary forces shaping microbial diversity in the human intestine. Cell (2006) 124(4):837–48. 10.1016/j.cell.2006.02.017 16497592

[4] Gilbert JA Blaser MJ Caporaso JG Jansson JK Lynch SV Knight R Current understanding of the human microbiome. Nat Med (2018) 24(4):392–400. 10.1038/nm.4517 29634682 PMC7043356

[5] Dominguez-Bello MG Costello EK Contreras M Magris M Hidalgo G Fierer N Delivery mode shapes the acquisition and structure of the initial microbiota across multiple body habitats in newborns. Proc Natl Acad Sci U S A (2010) 107(26):11971–5. 10.1073/pnas.1002601107 PMC2900693 20566857

[6] Yatsunenko T Rey FE Manary MJ Trehan I Dominguez-Bello MG Contreras M Human gut microbiome viewed across age and geography. Nature (2012) 486(7402):222–7. 10.1038/nature11053 PMC3376388 22699611

[7] Kundu P Blacher E Elinav E Pettersson S Our Gut Microbiome: The Evolving Inner Self. Cell (2017) 171(7):1481–93. 10.1016/j.cell.2017.11.024 29245010

[8] Bellone M Autoimmune Disease: Pathogenesis. Chichester, United Kingdom: eLS, John Wiley & Sons, Ltd (2015). 10.1002/9780470015902.a0001276.pub4

[9] Lambrecht BN Hammad H The immunology of the allergy epidemic and the hygiene hypothesis. Nat Immunol (2017) 18(10):1076–83. 10.1038/ni.3829 28926539

[10] Bellone M Brevi A Huber S Microbiota-Propelled T Helper 17 Cells in Inflammatory Diseases and Cancer. Microbiol Mol Biol Rev (2020) 84(2). 10.1128/MMBR.00064-19 PMC7062199 32132244

[11] Tanoue T Atarashi K Honda K Development and maintenance of intestinal regulatory T cells. Nat Rev Immunol (2016) 16(5):295–309. 10.1038/nri.2016.36 27087661

[12] Agus A Planchais J Sokol H Gut Microbiota Regulation of Tryptophan Metabolism in Health and Disease. Cell Host Microbe (2018) 23(6):716–24. 10.1016/j.chom.2018.05.003 29902437

[13] Durack J Lynch SV The gut microbiome: Relationships with disease and opportunities for therapy. J Exp Med (2019) 216(1):20–40. 10.1084/jem.20180448 30322864 PMC6314516

[14] Honda K Littman DR The microbiome in infectious disease and inflammation. Annu Rev Immunol (2012) 30:759–95. 10.1146/annurev-immunol-020711-074937 PMC4426968 22224764

[15] Ivanov II Honda K Intestinal commensal microbes as immune modulators. Cell Host Microbe (2012) 12(4):496–508. 10.1016/j.chom.2012.09.009 23084918 PMC3516493

[16] Wolk K Sabat R Interleukin-22: a novel T- and NK-cell derived cytokine that regulates the biology of tissue cells. Cytokine Growth Factor Rev (2006) 17(5):367–80. 10.1016/j.cytogfr.2006.09.001 17030002

[17] Hirota K Turner J Villa M Duarte JH Demengeot J Steinmetz OM Plasticity of TH17 cells in Peyer's patches is responsible for the induction of T cell–dependent IgA responses. Nat Immunol (2013) 14:372–9. 10.1038/ni.2552 PMC3672955 23475182

[18] Iwakura Y Ishigame H Saijo S Nakae S Functional specialization of interleukin-17 family members. Immunity (2011) 34(2):149–62. 10.1016/j.immuni.2011.02.012 21349428

[19] Gaffen SL Jain R Garg AV Cua DJ The IL-23-IL-17 immune axis: from mechanisms to therapeutic testing. Nat Rev Immunol (2014) 14(9):585–600. 10.1038/nri3707 25145755 PMC4281037

[20] Veldhoen M Interleukin 17 is a chief orchestrator of immunity. Nat Immunol (2017) 18(6):612–21. 10.1038/ni.3742 28518156

[21] Tilg H Zmora N Adolph TE Elinav E The intestinal microbiota fuelling metabolic inflammation. Nat Rev Immunol (2020) 20(1):40–54. 10.1038/s41577-019-0198-4 31388093

[22] Sato K Suematsu A Okamoto K Yamaguchi A Morishita Y Kadono Y Th17 functions as an osteoclastogenic helper T cell subset that links T cell activation and bone destruction. J Exp Med (2006) 203(12):2673–82. 10.1084/jem.20061775 PMC2118166 17088434

[23] de Aquino SG Abdollahi-Roodsaz S Koenders MI van de Loo FA Pruijn GJ Marijnissen RJ Periodontal pathogens directly promote autoimmune experimental arthritis by inducing a TLR2- and IL-1-driven Th17 response. J Immunol (2014) 192(9):4103–11. 10.4049/jimmunol.1301970 24683190

[24] Schneider C O’Leary CE von Moltke J Liang HE Ang QY Turnbaugh PJ A Metabolite-Triggered Tuft Cell-ILC2 Circuit Drives Small Intestinal Remodeling. Cell (2018) 174(2):271–84 e14. 10.1016/j.cell.2018.05.014 29887373 PMC6046262

[25] Ishigame H Kakuta S Nagai T Kadoki M Nambu A Komiyama Y Differential roles of interleukin-17A and -17F in host defense against mucoepithelial bacterial infection and allergic responses. Immunity (2009) 30(1):108–19. 10.1016/j.immuni.2008.11.009 19144317

[26] Song X Zhu S Shi P Liu Y Shi Y Levin SD IL-17RE is the functional receptor for IL-17C and mediates mucosal immunity to infection with intestinal pathogens. Nat Immunol (2011) 12(12):1151–8. 10.1038/ni.2155 21993849

[27] Reynolds JM Lee YH Shi Y Wang X Angkasekwinai P Nallaparaju KC Interleukin-17B Antagonizes Interleukin-25-Mediated Mucosal Inflammation. Immunity (2015) 42(4):692–703. 10.4049/jimmunol.1103014 25888259 PMC5811222

[28] Lee Y Clinton J Yao C Chang SH Interleukin-17D Promotes Pathogenicity During Infection by Suppressing CD8 T Cell Activity. Front Immunol (2019) 10:1172. 10.3389/fimmu.2019.01172 31244826 PMC6562898

[29] Wolf L Sapich S Honecker A Jungnickel C Seiler F Bischoff M IL-17A-mediated expression of epithelial IL-17C promotes inflammation during acute Pseudomonas aeruginosa pneumonia. Am J Physiol Lung Cell Mol Physiol (2016) 311(5):L1015–L22. 10.1152/ajplung.00158.2016 27694471

[30] Yang D Chen X Wang J Lou Q Lou Y Li L Dysregulated Lung Commensal Bacteria Drive Interleukin-17B Production to Promote Pulmonary Fibrosis through Their Outer Membrane Vesicles. Immunity (2019) 50(3):692–706 e7. 10.1016/j.immuni.2019.02.001 30824326

[31] Chen Y Chen Y Cao P Su W Zhan N Dong W Fusobacterium nucleatum facilitates ulcerative colitis through activating IL-17F signaling to NF-kappaB via the upregulation of CARD3 expression. J Pathol (2020) 250(2):170–82. 10.1002/path.5358 31610014

[32] Scher JU Sczesnak A Longman RS Segata N Ubeda C Bielski C Expansion of intestinal Prevotella copri correlates with enhanced susceptibility to arthritis. eLife (2013) 2:e01202. 10.7554/eLife.01202 24192039 PMC3816614

[33] Li Q Ma L Shen S Guo Y Cao Q Cai X Intestinal dysbacteriosis-induced IL-25 promotes development of HCC via alternative activation of macrophages in tumor microenvironment. J Exp Clin Cancer Res (2019) 38(1):303. 10.1186/s13046-019-1456-9 31296243 PMC6625119

[34] Song X Gao H Lin Y Yao Y Zhu S Wang J Alterations in the microbiota drive interleukin-17C production from intestinal epithelial cells to promote tumorigenesis. Immunity (2014) 40(1):140–52. 10.1016/j.immuni.2013.11.018 24412611

[35] Jungnickel C Schmidt LH Bittigkoffer L Wolf L Wolf A Ritzmann F IL-17C mediates the recruitment of tumor-associated neutrophils and lung tumor growth. Oncogene (2017) 36(29):4182–90. 10.1038/onc.2017.28 28346430

[36] Wu S Rhee KJ Albesiano E Rabizadeh S Wu X Yen HR A human colonic commensal promotes colon tumorigenesis via activation of T helper type 17 T cell responses. Nat Med (2009) 15(9):1016–22. 10.1038/nm.2015 PMC3034219 19701202

[37] Calcinotto A Brevi A Chesi M Ferrarese R Garcia Perez L Grioni M Microbiota-driven interleukin-17-producing cells and eosinophils synergize to accelerate multiple myeloma progression. Nat Commun (2018) 9(1):4832. 10.1038/s41467-018-07305-8 30510245 PMC6277390

[38] Maeda Y Kurakawa T Umemoto E Motooka D Ito Y Gotoh K Dysbiosis Contributes to Arthritis Development via Activation of Autoreactive T Cells in the Intestine. Arthritis Rheumatol (2016) 68(11):2646–61. 10.1002/art.39783 27333153

[39] Sospedra M Martin R Immunology of multiple sclerosis. Annu Rev Immunol (2005) 23:683–747. 10.1146/annurev.immunol.23.021704.115707 15771584

[40] Berer K Mues M Koutrolos M Rasbi ZA Boziki M Johner C Commensal microbiota and myelin autoantigen cooperate to trigger autoimmune demyelination. Nature (2011) 479(7374):538–41. 10.1038/nature10554 22031325

[41] Esplugues E Huber S Gagliani N Hauser AE Town T Wan YY Control of TH17 cells occurs in the small intestine. Nature (2011) 475(7357):514–8. 10.1038/nature10228 PMC3148838 21765430

[42] Duscha A Gisevius B Hirschberg S Yissachar N Stangl GI Eilers E Propionic Acid Shapes the Multiple Sclerosis Disease Course by an Immunomodulatory Mechanism. Cell (2020) 180(6):1067–80. 10.1016/j.cell.2020.02.035 32160527

[43] Zwicky P Unger S Becher B Targeting interleukin-17 in chronic inflammatory disease: A clinical perspective. J Exp Med (2020) 217(1). 10.1084/jem.20191123 PMC7037236 31727781

[44] Amatya N Garg AV Gaffen SL IL-17 Signaling: The Yin and the Yang. Trends Immunol (2017) 38(5):310–22. 10.1016/j.it.2017.01.006 PMC5411326 28254169

[45] Beringer A Miossec P Systemic effects of IL-17 in inflammatory arthritis. Nat Rev Rheumatol (2019) 15(8):491–501. 10.1038/s41584-019-0243-5 31227819

[46] Poore GD Kopylova E Zhu Q Carpenter C Fraraccio S Wandro S Microbiome analyses of blood and tissues suggest cancer diagnostic approach. Nature (2020) 579(7800):567–74. 10.1038/s41586-020-2095-1 PMC7500457 32214244

[47] Vitiello GA Miller G Targeting the interleukin-17 immune axis for cancer immunotherapy. J Exp Med (2020) 217(1). 10.1084/jem.20190456 PMC7037254 31727783

[48] Martin-Orozco N Muranski P Chung Y Yang XO Yamazaki T Lu S T helper 17 cells promote cytotoxic T cell activation in tumor immunity. Immunity (2009) 31(5):787–98. 10.1016/j.immuni.2009.09.014 PMC2787786 19879162

[49] Kryczek I Banerjee M Cheng P Vatan L Szeliga W Wei S Phenotype, distribution, generation, and functional and clinical relevance of Th17 cells in the human tumor environments. Blood (2009) 114(6):1141–9. 10.1182/blood-2009-03-208249 PMC2723011 19470694

[50] Sarnaik AA Yu B Yu D Morelli D Hall M Bogle D Extended dose ipilimumab with a peptide vaccine: immune correlates associated with clinical benefit in patients with resected high-risk stage IIIc/IV melanoma. Clin Cancer Res (2011) 17(4):896–906. 10.1158/1078-0432.CCR-10-2463 21106722 PMC3041838

[51] Xu M Pokrovskii M Ding Y Yi R Au C Harrison OJ c-MAF-dependent regulatory T cells mediate immunological tolerance to a gut pathobiont. Nature (2018) 554(7692):373–7. 10.1038/nature25500 PMC5814346 29414937

[52] Gomes AL Teijeiro A Buren S Tummala KS Yilmaz M Waisman A Metabolic Inflammation-Associated IL-17A Causes Non-alcoholic Steatohepatitis and Hepatocellular Carcinoma. Cancer Cell (2016) 30(1):161–75. 10.1016/j.ccell.2016.05.020 27411590

[53] McAllister F Bailey JM Alsina J Nirschl CJ Sharma R Fan H Oncogenic Kras activates a hematopoietic-to-epithelial IL-17 signaling axis in preinvasive pancreatic neoplasia. Cancer Cell (2014) 25(5):621–37. 10.1016/j.ccr.2014.03.014 PMC4072043 24823639

[54] Wang L Yi T Kortylewski M Pardoll DM Zeng D Yu H IL-17 can promote tumor growth through an IL-6-Stat3 signaling pathway. J Exp Med (2009) 206(7):1457–64. 10.1084/jem.20090207 PMC2715087 19564351

[55] Benevides L da Fonseca DM Donate PB Tiezzi DG De Carvalho DD de Andrade JM IL17 Promotes Mammary Tumor Progression by Changing the Behavior of Tumor Cells and Eliciting Tumorigenic Neutrophils Recruitment. Cancer Res (2015) 75(18):3788–99. 10.1158/0008-5472.CAN-15-0054 PMC8101363 26208902

[56] Coffelt SB Kersten K Doornebal CW Weiden J Vrijland K Hau CS IL-17-producing gammadelta T cells and neutrophils conspire to promote breast cancer metastasis. Nature (2015) 522(7556):345–8. 10.1038/nature14282 PMC4475637 25822788

[57] Chung AS Wu X Zhuang G Ngu H Kasman I Zhang J An interleukin-17-mediated paracrine network promotes tumor resistance to anti-angiogenic therapy. Nat Med (2013) 19(9):1114–23. 10.1038/nm.3291 23913124

[58] Li J Sung CY Lee N Ni Y Pihlajamaki J Panagiotou G Probiotics modulated gut microbiota suppresses hepatocellular carcinoma growth in mice. Proc Natl Acad Sci U S A (2016) 113(9):E1306–15. 10.1073/pnas.1518189113 PMC4780612 26884164

[59] Chesi M Robbiani DF Sebag M Chng WJ Affer M Tiedemann R AID-dependent activation of a MYC transgene induces multiple myeloma in a conditional mouse model of post-germinal center malignancies. Cancer Cell (2008) 13(2):167–80. 10.1016/j.ccr.2008.01.007 PMC2255064 18242516

[60] Calcinotto A Grioni M Jachetti E Curnis F Mondino A Parmiani G Targeting TNF-alpha to neoangiogenic vessels enhances lymphocyte infiltration in tumors and increases the therapeutic potential of immunotherapy. J Immunol (2012) 188(6):2687–94. 10.4049/jimmunol.1101877 22323546

[61] Prabhala RH Pelluru D Fulciniti M Prabhala HK Nanjappa P Song W Elevated IL-17 produced by TH17 cells promotes myeloma cell growth and inhibits immune function in multiple myeloma. Blood (2010) 115(26):5385–92. 10.1182/blood-2009-10-246660 PMC2902136 20395418

[62] Matson V Fessler J Bao R Chongsuwat T Zha Y Alegre ML The commensal microbiome is associated with anti-PD-1 efficacy in metastatic melanoma patients. Science (2018) 359(6371):104–8. 10.1126/science.aao3290 PMC6707353 29302014

[63] Routy B Le Chatelier E Derosa L Duong CPM Alou MT Daillere R Gut microbiome influences efficacy of PD-1-based immunotherapy against epithelial tumors. Science (2018) 359(6371):91–7. 10.1126/science.aan3706 29097494

[64] Gopalakrishnan V Spencer CN Nezi L Reuben A Andrews MC Karpinets TV Gut microbiome modulates response to anti-PD-1 immunotherapy in melanoma patients. Science (2018) 359(6371):97–103. 10.1126/science.aan4236 29097493 PMC5827966

[65] Rouvier E Luciani MF Mattei MG Denizot F Golstein P CTLA-8, cloned from an activated T cell, bearing AU-rich messenger RNA instability sequences, and homologous to a herpesvirus saimiri gene. J Immunol (1993) 150(12):5445–56. 8390535

[66] McGeachy MJ Cua DJ Gaffen SL The IL-17 Family of Cytokines in Health and Disease. Immunity (2019) 50(4):892–906. 10.1016/j.immuni.2019.03.021 30995505 PMC6474359

[67] Brembilla NC Senra L Boehncke WH The IL-17 Family of Cytokines in Psoriasis: IL-17A and Beyond. Front Immunol (2018) 9:1682. 10.3389/fimmu.2018.01682 30127781 PMC6088173

[68] Li X Bechara R Zhao J McGeachy MJ Gaffen SL IL-17 receptor-based signaling and implications for disease. Nat Immunol (2019) 20(12):1594–602. 10.1038/s41590-019-0514-y PMC6943935 31745337

[69] Qian Y Liu C Hartupee J Altuntas CZ Gulen MF Jane-Wit D The adaptor Act1 is required for interleukin 17-dependent signaling associated with autoimmune and inflammatory disease. Nat Immunol (2007) 8(3):247–56. 10.1038/ni1439 17277779

[70] Ogura H Murakami M Okuyama Y Tsuruoka M Kitabayashi C Kanamoto M Interleukin-17 promotes autoimmunity by triggering a positive-feedback loop via interleukin-6 induction. Immunity (2008) 29(4):628–36. 10.1016/j.immuni.2008.07.018 18848474

[71] Aggarwal S Gurney AL IL-17: prototype member of an emerging cytokine family. J Leukoc Biol (2002) 71(1):1–8. 10.1189/jlb.71.1.1 11781375

[72] Novatchkova M Leibbrandt A Werzowa J Neubuser A Eisenhaber F The STIR-domain superfamily in signal transduction, development and immunity. Trends Biochem Sci (2003) 28(5):226–9. 10.1016/S0968-0004(03)00067-7 12765832

[73] Wright JF Bennett F Li B Brooks J Luxenberg DP Whitters MJ The human IL-17F/IL-17A heterodimeric cytokine signals through the IL-17RA/IL-17RC receptor complex. J Immunol (2008) 181(4):2799–805. 10.4049/jimmunol.181.4.2799 18684971

[74] Wright JF Guo Y Quazi A Luxenberg DP Bennett F Ross JF Identification of an interleukin 17F/17A heterodimer in activated human CD4+ T cells. J Biol Chem (2007) 282(18):13447–55. 10.1074/jbc.M700499200 17355969

[75] Mellett M Atzei P Horgan A Hams E Floss T Wurst W Orphan receptor IL-17RD tunes IL-17A signalling and is required for neutrophilia. Nat Commun (2012) 3:1119. 10.1038/ncomms2127 23047677

[76] Chang SH Park H Dong C Act1 adaptor protein is an immediate and essential signaling component of interleukin-17 receptor. J Biol Chem (2006) 281(47):35603–7. 10.1074/jbc.C600256200 17035243

[77] Bulek K Liu C Swaidani S Wang L Page RC Gulen MF The inducible kinase IKKi is required for IL-17-dependent signaling associated with neutrophilia and pulmonary inflammation. Nat Immunol (2011) 12(9):844–52. 10.1038/ni.2080 PMC3282992 21822257

[78] Garg AV Ahmed M Vallejo AN Ma A Gaffen SL The deubiquitinase A20 mediates feedback inhibition of interleukin-17 receptor signaling. Sci Signal (2013) 6(278):ra44. 10.1126/scisignal.2003699 23737552 PMC4028484

[79] Zhong B Liu X Wang X Chang SH Liu X Wang A Negative regulation of IL-17-mediated signaling and inflammation by the ubiquitin-specific protease USP25. Nat Immunol (2012) 13(11):1110–7. 10.1038/ni.2427 PMC3477275 23042150

[80] Shi P Zhu S Lin Y Liu Y Liu Y Chen Z Persistent stimulation with interleukin-17 desensitizes cells through SCFbeta-TrCP-mediated degradation of Act1. Sci Signal (2011) 4(197):ra73. 10.1126/scisignal.2001653 22045853

[81] Liu C Qian W Qian Y Giltiay NV Lu Y Swaidani S Act1, a U-box E3 ubiquitin ligase for IL-17 signaling. Sci Signal (2009) 2(92):ra63. 10.1126/scisignal.2000382 19825828 PMC3182834

[82] Ruddy MJ Wong GC Liu XK Yamamoto H Kasayama S Kirkwood KL Functional cooperation between interleukin-17 and tumor necrosis factor-alpha is mediated by CCAAT/enhancer-binding protein family members. J Biol Chem (2004) 279(4):2559–67. 10.1074/jbc.M308809200 14600152

[83] Song X Dai D He X Zhu S Yao Y Gao H Growth Factor FGF2 Cooperates with Interleukin-17 to Repair Intestinal Epithelial Damage. Immunity (2015) 43(3):488–501. 10.1016/j.immuni.2015.06.024 26320657

[84] Verma AH Richardson JP Zhou C Coleman BM Moyes DL Ho J Oral epithelial cells orchestrate innate type 17 responses to Candida albicans through the virulence factor candidalysin. Sci Immunol (2017) 2(17). 10.1126/sciimmunol.aam8834 PMC5881387 29101209

[85] Chen X Cai G Liu C Zhao J Gu C Wu L IL-17R-EGFR axis links wound healing to tumorigenesis in Lrig1(+) stem cells. J Exp Med (2019) 216(1):195–214. 10.1084/jem.20171849 30578323 PMC6314525

[86] Wu L Chen X Zhao J Martin B Zepp JA Ko JS A novel IL-17 signaling pathway controlling keratinocyte proliferation and tumorigenesis via the TRAF4-ERK5 axis. J Exp Med (2015) 212(10):1571–87. 10.1084/jem.20150204 PMC4577838 26347473

[87] Shao X Chen S Yang D Cao M Yao Y Wu Z FGF2 cooperates with IL-17 to promote autoimmune inflammation. Sci Rep (2017) 7(1):7024. 10.1038/s41598-017-07597-8 28765647 PMC5539112

[88] Kang Z Wang C Zepp J Wu L Sun K Zhao J Act1 mediates IL-17-induced EAE pathogenesis selectively in NG2+ glial cells. Nat Neurosci (2013) 16(10):1401–8. 10.1038/nn.3505 PMC4106025 23995070

[89] Wang C Zhang CJ Martin BN Bulek K Kang Z Zhao J IL-17 induced NOTCH1 activation in oligodendrocyte progenitor cells enhances proliferation and inflammatory gene expression. Nat Commun (2017) 8:15508. 10.1038/ncomms15508 28561022 PMC5460031

[90] Langley RG Elewski BE Lebwohl M Reich K Griffiths CE Papp K Secukinumab in plaque psoriasis–results of two phase 3 trials. N Engl J Med (2014) 371(4):326–38. 10.1056/NEJMoa1314258 25007392

[91] Nies JF Panzer U IL-17C/IL-17RE: Emergence of a Unique Axis in TH17 Biology. Front Immunol (2020) 11:341. 10.3389/fimmu.2020.00341 32174926 PMC7054382

[92] Chang SH Dong C IL-17F: regulation, signaling and function in inflammation. Cytokine (2009) 46(1):7–11. 10.1016/j.cyto.2008.12.024 19233684 PMC2663007

[93] Bie Q Jin C Zhang B Dong H IL-17B: A new area of study in the IL-17 family. Mol Immunol (2017) 90:50–6. 10.1016/j.molimm.2017.07.004 28704706

[94] Patel DD Kuchroo VK Th17 Cell Pathway in Human Immunity: Lessons from Genetics and Therapeutic Interventions. Immunity (2015) 43(6):1040–51. 10.1016/j.immuni.2015.12.003 26682981

[95] Gerhardt S Abbott WM Hargreaves D Pauptit RA Davies RA Needham MR Structure of IL-17A in complex with a potent, fully human neutralizing antibody. J Mol Biol (2009) 394(5):905–21. 10.1016/j.jmb.2009.10.008 19835883

[96] Starnes T Broxmeyer HE Robertson MJ Hromas R Cutting edge: IL-17D, a novel member of the IL-17 family, stimulates cytokine production and inhibits hemopoiesis. J Immunol (2002) 169(2):642–6. 10.4049/jimmunol.169.2.642 12097364

[97] Akimzhanov AM Yang XO Dong C Chromatin remodeling of interleukin-17 (IL-17)-IL-17F cytokine gene locus during inflammatory helper T cell differentiation. J Biol Chem (2007) 282(9):5969–72. 10.1074/jbc.C600322200 17218320

[98] Okada S Puel A Casanova JL Kobayashi M Chronic mucocutaneous candidiasis disease associated with inborn errors of IL-17 immunity. Clin Transl Immunol (2016) 5(12):e114. 10.1038/cti.2016.71 PMC5192062 28090315

[99] Gomez-Rodriguez J Sahu N Handon R Davidson TS Anderson SM Kirby MR Differential expression of interleukin-17A and -17F is coupled to T cell receptor signaling via inducible T cell kinase. Immunity (2009) 31(4):587–97. 10.1016/j.immuni.2009.07.009 PMC2767186 19818650

[100] Starnes T Robertson MJ Sledge G Kelich S Nakshatri H Broxmeyer HE Cutting edge: IL-17F, a novel cytokine selectively expressed in activated T cells and monocytes, regulates angiogenesis and endothelial cell cytokine production. J Immunol (2001) 167(8):4137–40. 10.4049/jimmunol.167.8.4137 11591732

[101] Kawaguchi M Onuchic LF Li XD Essayan DM Schroeder J Xiao HQ Identification of a novel cytokine, ML-1, and its expression in subjects with asthma. J Immunol (2001) 167(8):4430–5. 10.4049/jimmunol.1201505 11591768

[102] Chang SH Dong C A novel heterodimeric cytokine consisting of IL-17 and IL-17F regulates inflammatory responses. Cell Res (2007) 17(5):435–40. 10.1038/cr.2007.35 17452998

[103] Hot A Miossec P Effects of interleukin (IL)-17A and IL-17F in human rheumatoid arthritis synoviocytes. Ann Rheum Dis (2011) 70(5):727–32. 10.1136/ard.2010.143768 21345813

[104] Goepfert A Lehmann S Blank J Kolbinger F Rondeau JM Structural Analysis Reveals that the Cytokine IL-17F Forms a Homodimeric Complex with Receptor IL-17RC to Drive IL-17RA-Independent Signaling. Immunity (2020) 52(3):499–512 e5. 10.1016/j.immuni.2020.02.004 32187518

[105] Tang C Kakuta S Shimizu K Kadoki M Kamiya T Shimazu T Suppression of IL-17F, but not of IL-17A, provides protection against colitis by inducing Treg cells through modification of the intestinal microbiota. Nat Immunol (2018) 19(7):755–65. 10.1038/s41590-018-0134-y 29915298

[106] Kamiya T Tang C Kadoki M Oshima K Hattori M Saijo S beta-Glucans in food modify colonic microflora by inducing antimicrobial protein, calprotectin, in a Dectin-1-induced-IL-17F-dependent manner. Mucosal Immunol (2018) 11(3):763–73. 10.1038/mi.2017.86 29068000

[107] Ramirez-Carrozzi V Sambandam A Luis E Lin Z Jeet S Lesch J IL-17C regulates the innate immune function of epithelial cells in an autocrine manner. Nat Immunol (2011) 12(12):1159–66. 10.1038/ni.2156 21993848

[108] Kolls JK Linden A Interleukin-17 family members and inflammation. Immunity (2004) 21(4):467–76. 10.1016/j.immuni.2004.08.018 15485625

[109] Reynolds JM Martinez GJ Nallaparaju KC Chang SH Wang YH Dong C Cutting edge: regulation of intestinal inflammation and barrier function by IL-17C. J Immunol (2012) 189(9):4226–30. 10.1074/jbc.M910228199 PMC3478486 23024280

[110] Chang SH Reynolds JM Pappu BP Chen G Martinez GJ Dong C Interleukin-17C promotes Th17 cell responses and autoimmune disease via interleukin-17 receptor E. Immunity (2011) 35(4):611–21. 10.1016/j.immuni.2011.09.010 PMC5800502 21982598

[111] Johnston A Fritz Y Dawes SM Diaconu D Al-Attar PM Guzman AM Keratinocyte overexpression of IL-17C promotes psoriasiform skin inflammation. J Immunol (2013) 190(5):2252–62. 10.4049/jimmunol.1201505 PMC3577967 23359500

[112] Martin DA Towne JE Kricorian G Klekotka P Gudjonsson JE Krueger JG The emerging role of IL-17 in the pathogenesis of psoriasis: preclinical and clinical findings. J Invest Dermatol (2013) 133(1):17–26. 10.1038/jid.2012.194 22673731 PMC3568997

[113] Smith E Prasad KM Butcher M Dobrian A Kolls JK Ley K Blockade of interleukin-17A results in reduced atherosclerosis in apolipoprotein E-deficient mice. Circulation (2010) 121(15):1746–55. 10.1161/CIRCULATIONAHA.109.924886 PMC2929562 20368519

[114] Butcher MJ Waseem TC Galkina EV Smooth Muscle Cell-Derived Interleukin-17C Plays an Atherogenic Role via the Recruitment of Proinflammatory Interleukin-17A+ T Cells to the Aorta. Arterioscler Thromb Vasc Biol (2016) 36(8):1496–506. 10.1161/ATVBAHA.116.307892 PMC5242324 27365405

[115] Butcher MJ Gjurich BN Phillips T Galkina EV The IL-17A/IL-17RA axis plays a proatherogenic role via the regulation of aortic myeloid cell recruitment. Circ Res (2012) 110(5):675–87. 10.1161/CIRCRESAHA.111.261784 PMC3337709 22302786

[116] Krohn S Nies JF Kapffer S Schmidt T Riedel JH Kaffke A IL-17C/IL-17 Receptor E Signaling in CD4(+) T Cells Promotes TH17 Cell-Driven Glomerular Inflammation. J Am Soc Nephrol (2018) 29(4):1210–22. 10.1681/ASN.2017090949 PMC5875959 29483158

[117] Yao Z Painter SL Fanslow WC Ulrich D Macduff BM Spriggs MK Human IL-17: a novel cytokine derived from T cells. J Immunol (1995) 155(12):5483–6. 7499828

[118] Li H Chen J Huang A Stinson J Heldens S Foster J Cloning and characterization of IL-17B and IL-17C, two new members of the IL-17 cytokine family. Proc Natl Acad Sci U S A (2000) 97(2):773–8. 10.1073/pnas.97.2.773 PMC15406 10639155

[119] Shi Y Ullrich SJ Zhang J Connolly K Grzegorzewski KJ Barber MC A novel cytokine receptor-ligand pair. Identification, molecular characterization, and in vivo immunomodulatory activity. J Biol Chem (2000) 275(25):19167–76. 10.1074/jbc.M910228199 10749887

[120] Lee J Ho WH Maruoka M Corpuz RT Baldwin DT Foster JS IL-17E, a novel proinflammatory ligand for the IL-17 receptor homolog IL-17Rh1. J Biol Chem (2001) 276(2):1660–4. 10.1074/jbc.M008289200 11058597

[121] Al-Samadi A Moossavi S Salem A Sotoudeh M Tuovinen SM Konttinen YT Distinctive expression pattern of interleukin-17 cytokine family members in colorectal cancer. Tumour Biol (2016) 37(2):1609–15. 10.1007/s13277-015-3941-x 26304506

[122] Ferretti E Ponzoni M Doglioni C Pistoia V IL-17 superfamily cytokines modulate normal germinal center B cell migration. J Leukoc Biol (2016) 100(5):913–8. 10.1189/jlb.1VMR0216-096RR 27566830

[123] Ryan AW Thornton JM Brophy K Daly JS McLoughlin RM O’Morain C Chromosome 5q candidate genes in coeliac disease: genetic variation at IL4, IL5, IL9, IL13, IL17B and NR3C1. Tissue Antigens (2005) 65(2):150–5. 10.1111/j.1399-0039.2005.00354.x 15713213

[124] Robak E Kulczycka-Siennicka L Gerlicz Z Kierstan M Korycka-Wolowiec A Sysa-Jedrzejowska A Correlations between concentrations of interleukin (IL)-17A, IL-17B and IL-17F, and endothelial cells and proangiogenic cytokines in systemic lupus erythematosus patients. Eur Cytokine Netw (2013) 24(1):60–8. 10.1684/ecn.2013.0330 23661335

[125] Zhou J Ren L Chen D Lin X Huang S Yin Y IL-17B is elevated in patients with pneumonia and mediates IL-8 production in bronchial epithelial cells. Clin Immunol (2017) 175:91–8. 10.1016/j.clim.2016.12.008 28039016

[126] Kouri VP Olkkonen J Ainola M Li TF Bjorkman L Konttinen YT Neutrophils produce interleukin-17B in rheumatoid synovial tissue. Rheumatol (Oxford) (2014) 53(1):39–47. 10.1093/rheumatology/ket309 24056520

[127] Yamaguchi Y Fujio K Shoda H Okamoto A Tsuno NH Takahashi K IL-17B and IL-17C are associated with TNF-alpha production and contribute to the exacerbation of inflammatory arthritis. J Immunol (2007) 179(10):7128–36. 10.4049/jimmunol.179.10.7128 17982105

[128] Bie Q Sun C Gong A Li C Su Z Zheng D Non-tumor tissue derived interleukin-17B activates IL-17RB/AKT/beta-catenin pathway to enhance the stemness of gastric cancer. Sci Rep (2016) 6:25447. 10.1038/srep25447 27146881 PMC4857095

[129] Sanders AJ Guo X Mason MD Jiang WG IL-17B Can Impact on Endothelial Cellular Traits Linked to Tumour Angiogenesis. J Oncol (2010) 2010:817375. 10.1155/2010/817375 20467469 PMC2866247

[130] Buning C Genschel J Weltrich R Lochs H Schmidt H The interleukin-25 gene located in the inflammatory bowel disease (IBD) 4 region: no association with inflammatory bowel disease. Eur J Immunogenet (2003) 30(5):329–33. 10.1046/j.1365-2370.2003.00411.x 14641539

[131] Liu Y Shao Z Shangguan G Bie Q Zhang B Biological Properties and the Role of IL-25 in Disease Pathogenesis. J Immunol Res (2018) 2018:6519465. 10.1155/2018/6519465 30345318 PMC6174801

[132] von Moltke J Ji M Liang HE Locksley RM Tuft-cell-derived IL-25 regulates an intestinal ILC2-epithelial response circuit. Nature (2016) 529(7585):221–5. 10.1038/nature16161 PMC4830391 26675736

[133] Fort MM Cheung J Yen D Li J Zurawski SM Lo S IL-25 induces IL-4, IL-5, and IL-13 and Th2-associated pathologies in vivo. Immunity (2001) 15(6):985–95. 10.1016/S1074-7613(01)00243-6 11754819

[134] Angkasekwinai P Park H Wang YH Wang YH Chang SH Corry DB Interleukin 25 promotes the initiation of proallergic type 2 responses. J Exp Med (2007) 204(7):1509–17. 10.1084/jem.20061675 PMC2118650 17562814

[135] Kang CM Jang AS Ahn MH Shin JA Kim JH Choi YS Interleukin-25 and interleukin-13 production by alveolar macrophages in response to particles. Am J Respir Cell Mol Biol (2005) 33(3):290–6. 10.1165/rcmb.2005-0003OC 15961726

[136] Wang WB Yen ML Liu KJ Hsu PJ Lin MH Chen PM Interleukin-25 Mediates Transcriptional Control of PD-L1 via STAT3 in Multipotent Human Mesenchymal Stromal Cells (hMSCs) to Suppress Th17 Responses. Stem Cell Rep (2015) 5(3):392–404. 10.1016/j.stemcr.2015.07.013 PMC4618596 26321145

[137] Ikeda K Nakajima H Suzuki K Kagami S Hirose K Suto A Mast cells produce interleukin-25 upon Fc epsilon RI-mediated activation. Blood (2003) 101(9):3594–6. 10.1182/blood-2002-09-2817 12511410

[138] Kleinschek MA Owyang AM Joyce-Shaikh B Langrish CL Chen Y Gorman DM IL-25 regulates Th17 function in autoimmune inflammation. J Exp Med (2007) 204(1):161–70. 10.1084/jem.20061738 PMC2118427 17200411

[139] Letuve S Lajoie-Kadoch S Audusseau S Rothenberg ME Fiset PO Ludwig MS IL-17E upregulates the expression of proinflammatory cytokines in lung fibroblasts. J Allergy Clin Immunol (2006) 117(3):590–6. 10.1016/j.jaci.2005.10.025 16522458

[140] Sonobe Y Takeuchi H Kataoka K Li H Jin S Mimuro M Interleukin-25 expressed by brain capillary endothelial cells maintains blood-brain barrier function in a protein kinase Cepsilon-dependent manner. J Biol Chem (2009) 284(46):31834–42. 10.1074/jbc.M109.025940 PMC2797254 19776017

[141] Price AE Liang HE Sullivan BM Reinhardt RL Eisley CJ Erle DJ Systemically dispersed innate IL-13-expressing cells in type 2 immunity. Proc Natl Acad Sci U S A (2010) 107(25):11489–94. 10.1073/pnas.1003988107 PMC2895098 20534524

[142] Neill DR Wong SH Bellosi A Flynn RJ Daly M Langford TK Nuocytes represent a new innate effector leukocyte that mediates type-2 immunity. Nature (2010) 464(7293):1367–70. 10.1038/nature08900 PMC2862165 20200518

[143] Terashima A Watarai H Inoue S Sekine E Nakagawa R Hase K A novel subset of mouse NKT cells bearing the IL-17 receptor B responds to IL-25 and contributes to airway hyperreactivity. J Exp Med (2008) 205(12):2727–33. 10.1084/jem.20080698 PMC2585837 19015310

[144] Maezawa Y Nakajima H Suzuki K Tamachi T Ikeda K Inoue J Involvement of TNF receptor-associated factor 6 in IL-25 receptor signaling. J Immunol (2006) 176(2):1013–8. 10.4049/jimmunol.176.2.1013 16393988

[145] Swaidani S Bulek K Kang Z Gulen MF Liu C Yin W T cell-derived Act1 is necessary for IL-25-mediated Th2 responses and allergic airway inflammation. J Immunol (2011) 187(6):3155–64. 10.4049/jimmunol.1002790 PMC3666175 21856933

[146] Beale J Jayaraman A Jackson DJ Macintyre JDR Edwards MR Walton RP Rhinovirus-induced IL-25 in asthma exacerbation drives type 2 immunity and allergic pulmonary inflammation. Sci Transl Med (2014) 6(256):256ra134. 10.1126/scitranslmed.3009124 PMC4246061 25273095

[147] Tamachi T Maezawa Y Ikeda K Kagami S Hatano M Seto Y IL-25 enhances allergic airway inflammation by amplifying a TH2 cell-dependent pathway in mice. J Allergy Clin Immunol (2006) 118(3):606–14. 10.1016/j.jaci.2006.04.051 16950278

[148] Ramirez-Carrozzi V Ota N Sambandam A Wong K Hackney J Martinez-Martin N Cutting Edge: IL-17B Uses IL-17RA and IL-17RB to Induce Type 2 Inflammation from Human Lymphocytes. J Immunol (2019) 202(7):1935–41. 10.4049/jimmunol.1800696 30770417

[149] Su J Chen T Ji XY Liu C Yadav PK Wu R IL-25 downregulates Th1/Th17 immune response in an IL-10-dependent manner in inflammatory bowel disease. Inflammation Bowel Dis (2013) 19(4):720–8. 10.1097/MIB.0b013e3182802a76 23429464

[150] Benatar T Cao MY Lee Y Lightfoot J Feng N Gu X IL-17E, a proinflammatory cytokine, has antitumor efficacy against several tumor types in vivo. Cancer Immunol Immunother (2010) 59(6):805–17. 10.1007/s00262-009-0802-8 PMC11030851 20012860

[151] Han Q Das S Hirano M Holland SJ McCurley N Guo P Characterization of Lamprey IL-17 Family Members and Their Receptors. J Immunol (2015) 195(11):5440–51. 10.4049/jimmunol.1500892 PMC4655163 26491201

[152] Saddawi-Konefka R Seelige R Gross ET Levy E Searles SC Washington A Jr. Nrf2 Induces IL-17D to Mediate Tumor and Virus Surveillance. Cell Rep (2016) 16(9):2348–58. 10.1016/j.celrep.2016.07.075 PMC5007173 27545889

[153] Seelige R Washington A Jr. Bui JD The ancient cytokine IL-17D is regulated by Nrf2 and mediates tumor and virus surveillance. Cytokine (2017) 91:10–2. 10.1016/j.cyto.2016.11.017 PMC5316352 27940089

[154] Stamp LK Easson A Lehnigk U Highton J Hessian PA Different T cell subsets in the nodule and synovial membrane: absence of interleukin-17A in rheumatoid nodules. Arthritis Rheumatol (2008) 58(6):1601–8. 10.1002/art.23455 18512780

[155] Johansen C Usher PA Kjellerup RB Lundsgaard D Iversen L Kragballe K Characterization of the interleukin-17 isoforms and receptors in lesional psoriatic skin. Br J Dermatol (2009) 160(2):319–24. 10.1111/j.1365-2133.2008.08902.x 19016708

[156] Hueber W Sands BE Lewitzky S Vandemeulebroecke M Reinisch W Higgins PD Secukinumab, a human anti-IL-17A monoclonal antibody, for moderate to severe Crohn’s disease: unexpected results of a randomised, double-blind placebo-controlled trial. Gut (2012) 61(12):1693–700. 10.1136/gutjnl-2011-301668 PMC4902107 22595313

[157] Lopez-Ferrer A Vilarrasa E Puig L Secukinumab (AIN457) for the treatment of psoriasis. Expert Rev Clin Immunol (2015) 11(11):1177–88. 10.1586/1744666X.2015.1095092 26428036

[158] Conti HR Whibley N Coleman BM Garg AV Jaycox JR Gaffen SL Signaling through IL-17C/IL-17RE is dispensable for immunity to systemic, oral and cutaneous candidiasis. PloS One (2015) 10(4):e0122807. 10.1371/journal.pone.0122807 25849644 PMC4388490

[159] Fallon PG Ballantyne SJ Mangan NE Barlow JL Dasvarma A Hewett DR Identification of an interleukin (IL)-25-dependent cell population that provides IL-4, IL-5, and IL-13 at the onset of helminth expulsion. J Exp Med (2006) 203(4):1105–16. 10.1084/jem.20051615 PMC2118283 16606668

[160] Owyang AM Zaph C Wilson EH Guild KJ McClanahan T Miller HR Interleukin 25 regulates type 2 cytokine-dependent immunity and limits chronic inflammation in the gastrointestinal tract. J Exp Med (2006) 203(4):843–9. 10.1084/jem.20051496 PMC1800834 16606667

[161] Zaph C Du Y Saenz SA Nair MG Perrigoue JG Taylor BC Commensal-dependent expression of IL-25 regulates the IL-23-IL-17 axis in the intestine. J Exp Med (2008) 205(10):2191–8. 10.1084/jem.20080720 PMC2556798 18762568

[162] Sawa S Lochner M Satoh-Takayama N Dulauroy S Berard M Kleinschek M RORgammat+ innate lymphoid cells regulate intestinal homeostasis by integrating negative signals from the symbiotic microbiota. Nat Immunol (2011) 12(4):320–6. 10.1038/ni.2002 21336274

[163] Hammond M Clark AB Cahn AP Chilvers ER Fraser WD Livermore DM The Efficacy and Mechanism Evaluation of Treating Idiopathic Pulmonary fibrosis with the Addition of Co-trimoxazole (EME-TIPAC): study protocol for a randomised controlled trial. Trials (2018) 19(1):89. 10.1186/s13063-018-2453-6 29402332 PMC5800095

[164] Macaluso C Maritano Furcada J Alzaher O Chaube R Chua F Wells AU The potential impact of azithromycin in idiopathic pulmonary fibrosis. Eur Respir J (2019) 53(2). 10.1183/13993003.00628-2018 30442715

[165] O’Sullivan T Saddawi-Konefka R Gross E Tran M Mayfield SP Ikeda H Interleukin-17D mediates tumor rejection through recruitment of natural killer cells. Cell Rep (2014) 7(4):989–98. 10.1016/j.celrep.2014.03.073 PMC4084720 24794441

[166] Prabhala RH Fulciniti M Pelluru D Rashid N Nigroiu A Nanjappa P Targeting IL-17A in multiple myeloma: a potential novel therapeutic approach in myeloma. Leukemia (2016) 30(2):379–89. 10.1038/leu.2015.228 PMC4740263 26293646

[167] Furuta S Jeng YM Zhou L Huang L Kuhn I Bissell MJ IL-25 causes apoptosis of IL-25R-expressing breast cancer cells without toxicity to nonmalignant cells. Sci Transl Med (2011) 3(78):78ra31. 10.1126/scitranslmed.3001374 PMC3199022 21490275

[168] Yin SY Jian FY Chen YH Chien SC Hsieh MC Hsiao PW Induction of IL-25 secretion from tumour-associated fibroblasts suppresses mammary tumour metastasis. Nat Commun (2016) 7:11311. 10.1038/ncomms11909 27089063 PMC4837478

[169] Cheng J Gu CJ Zhang B Xie F Yuan MM Li MQ Cisplatin inhibits the growth, migration and invasion of cervical cancer cells by down-regulating IL-17E/IL-17RB. Int J Clin Exp Pathol (2017) 10(9):9341–51. PMC6965941 31966806

[170] Huang CK Yang CY Jeng YM Chen CL Wu HH Chang YC Autocrine/paracrine mechanism of interleukin-17B receptor promotes breast tumorigenesis through NF-kappaB-mediated antiapoptotic pathway. Oncogene (2014) 33(23):2968–77. 10.1038/onc.2013.268 23851503

[171] Wu HH Hwang-Verslues WW Lee WH Huang CK Wei PC Chen CL Targeting IL-17B-IL-17RB signaling with an anti-IL-17RB antibody blocks pancreatic cancer metastasis by silencing multiple chemokines. J Exp Med (2015) 212(3):333–49. 10.1084/jem.20141702 PMC4354366 25732306

[172] Bie Q Zhang B Sun C Ji X Barnie PA Qi C IL-17B activated mesenchymal stem cells enhance proliferation and migration of gastric cancer cells. Oncotarget (2017) 8(12):18914–23. 10.18632/oncotarget.14835 PMC5386657 28145881

[173] Ren L Xu Y Liu C Wang S Qin G IL-17RB enhances thyroid cancer cell invasion and metastasis via ERK1/2 pathway-mediated MMP-9 expression. Mol Immunol (2017) 90:126–35. 10.1016/j.molimm.2017.06.034 28715683

[174] Guo HZ Niu LT Qiang WT Chen J Wang J Yang H Leukemic IL-17RB signaling regulates leukemic survival and chemoresistance. FASEB J (2019) 33(8):9565–76. 10.1096/fj.201900099R 31136196

[175] Liao R Sun J Wu H Yi Y Wang JX He HW High expression of IL-17 and IL-17RE associate with poor prognosis of hepatocellular carcinoma. J Exp Clin Cancer Res (2013) 32:3. 10.1186/1756-9966-32-3 23305119 PMC3621615

[176] Brennan CA Garrett WS Fusobacterium nucleatum - symbiont, opportunist and oncobacterium. Nat Rev Microbiol (2019) 17(3):156–66. 10.1038/s41579-018-0129-6 PMC6589823 30546113

[177] Vandeghinste N Klattig J Jagerschmidt C Lavazais S Marsais F Haas JD Neutralization of IL-17C Reduces Skin Inflammation in Mouse Models of Psoriasis and Atopic Dermatitis. J Invest Dermatol (2018) 138(7):1555–63. 10.1016/j.jid.2018.01.036 29474945

[178] Cammarota G Ianiro G Kelly CR Mullish BH Allegretti JR Kassam Z International consensus conference on stool banking for faecal microbiota transplantation in clinical practice. Gut (2019) 68(12):2111–21. 10.1136/gutjnl-2019-319548 PMC6872442 31563878

[179] Lynch SV Ng SC Shanahan F Tilg H Translating the gut microbiome: ready for the clinic? Nat Rev Gastroenterol Hepatol (2019) 16(11):656–61. 10.1038/s41575-019-0204-0 31562390

[180] Kolodziejczyk AA Zheng D Elinav E Diet-microbiota interactions and personalized nutrition. Nat Rev Microbiol (2019) 17(12):742–53. 10.1038/s41579-019-0256-8 31541197

[181] Suez J Zmora N Zilberman-Schapira G Mor U Dori-Bachash M Bashiardes S Post-Antibiotic Gut Mucosal Microbiome Reconstitution Is Impaired by Probiotics and Improved by Autologous FMT. Cell (2018) 174(6):1406–23. 10.1016/j.cell.2018.08.047 30193113

[182] Skelly AN Sato Y Kearney S Honda K Mining the microbiota for microbial and metabolite-based immunotherapies. Nat Rev Immunol (2019) 19(5):305–23. 10.1038/s41577-019-0144-5 30858494

[183] Elinav E Garrett WS Trinchieri G Wargo J The cancer microbiome. Nat Rev Cancer (2019) 19(7):371–6. 10.1038/s41568-019-0155-3 PMC6700740 31186547

[184] Helmink BA Khan MAW Hermann A Gopalakrishnan V Wargo JA The microbiome, cancer, and cancer therapy. Nat Med (2019) 25(3):377–88. 10.1038/s41591-019-0377-7 30842679

[185] Zitvogel L Daillere R Roberti MP Routy B Kroemer G Anticancer effects of the microbiome and its products. Nat Rev Microbiol (2017) 15:465–77. 10.1038/nrmicro.2017.44 28529325

[186] Ritchlin CT Kavanaugh A Merola JF Schett G Scher JU Warren RB Bimekizumab in patients with active psoriatic arthritis: results from a 48-week, randomised, double-blind, placebo-controlled, dose-ranging phase 2b trial. Lancet (2020) 395(10222):427–40. 10.1016/S0140-6736(19)33161-7 32035552

[187] Ma Q Role of nrf2 in oxidative stress and toxicity. Annu Rev Pharmacol Toxicol (2013) 53:401–26. 10.1146/annurev-pharmtox-011112-140320 PMC4680839 23294312

